# Complement inhibition decreases early fibrogenic events in the lung of septic baboons

**DOI:** 10.1111/jcmm.12667

**Published:** 2015-09-03

**Authors:** Robert Silasi-Mansat, Hua Zhu, Constantin Georgescu, Narcis Popescu, Ravi S Keshari, Glenn Peer, Cristina Lupu, Fletcher B Taylor, Heloise Anne Pereira, Gary Kinasewitz, John D Lambris, Florea Lupu

**Affiliations:** a Programs in Cardiovascular Biology, Oklahoma Medical Research FoundationOklahoma City, OK, USA; b Programs in Clinical Immunology, Oklahoma Medical Research FoundationOklahoma City, OK, USA; c Department of Medicine, Pulmonary and Critical Care Division, Oklahoma University Health Sciences CenterOklahoma City, OK, USA; d Department of Pathology, Oklahoma University Health Sciences CenterOklahoma City, OK, USA; e Department of Pharmaceutical Sciences, Oklahoma University Health Sciences CenterOklahoma City, OK, USA; f Department of Cell Biology, Oklahoma University Health Sciences CenterOklahoma City, OK, USA; g Department of Pathology and Laboratory Medicine, School of Medicine, University of PennsylvaniaPhiladelphia, PA, USA

**Keywords:** lung, organ failure, sepsis, complement, fibrosis

## Abstract

Acute respiratory distress syndrome (ARDS) induced by severe sepsis can trigger persistent inflammation and fibrosis. We have shown that experimental sepsis in baboons recapitulates ARDS progression in humans, including chronic inflammation and long-lasting fibrosis in the lung. Complement activation products may contribute to the fibroproliferative response, suggesting that complement inhibitors are potential therapeutic agents. We have been suggested that treatment of septic baboons with compstatin, a C3 convertase inhibitor protects against ARDS-induced fibroproliferation. Baboons challenged with 10^9^ cfu/kg (LD50) live *E. coli* by intravenous infusion were treated or not with compstatin at the time of challenge or 5 hrs thereafter. Changes in the fibroproliferative response at 24 hrs post-challenge were analysed at both transcript and protein levels. Gene expression analysis showed that sepsis induced fibrotic responses in the lung as early as 24 hrs post-bacterial challenge. Immunochemical and biochemical analysis revealed enhanced collagen synthesis, induction of profibrotic factors and increased cell recruitment and proliferation. Specific inhibition of complement with compstatin down-regulated sepsis-induced fibrosis genes, including transforming growth factor-beta (TGF-β), connective tissue growth factor (CTGF), tissue inhibitor of metalloproteinase 1 (TIMP1), various collagens and chemokines responsible for fibrocyte recruitment (*e.g*. chemokine (C-C motif) ligand 2 (CCL2) and 12 (CCL12)). Compstatin decreased the accumulation of myofibroblasts and proliferating cells, reduced the production of fibrosis mediators (TGF-β, phospho-Smad-2 and CTGF) and inhibited collagen deposition. Our data demonstrate that complement inhibition effectively attenuates collagen deposition and fibrotic responses in the lung after severe sepsis. Inhibiting complement could prove an attractive strategy for preventing sepsis-induced fibrosis of the lung.

## Introduction

Severe sepsis is the most common cause of the acute respiratory distress syndrome (ARDS), a form of microvascular lung injury that produces acute hypoxemic respiratory failure 1. Clinical and experimental evidence indicate that ARDS begins as an acute inflammatory lung injury, which quickly progresses to a fibroproliferative phase in which collagen deposition contributes to respiratory failure and prolonged ventilator dependence 2–5. Fibrogenesis begins within the first 24 hrs 3 and may resolve if the patient recovers, or it may progress to extensive and persistent fibrosis 5,6. Progressive pulmonary fibrosis can be the direct cause of respiratory death in up to 40% of patients and indirectly can contribute to death because of nosocomial infections and progressive multiple organ failure in up to 70% of patients who die from ARDS. Even in ARDS survivors, pulmonary fibrosis may lead to persistent impairment in pulmonary function and consequent morbidity. Thus, fibrosis is an important contributor to the clinical outcome and a potential therapeutic target in ARDS. Currently, there is no specific treatment for post-ARDS fibrosis other than supportive therapy, and therefore new molecular markers and therapeutic targets for this disabling and ultimately lethal disease are critically needed.

Clinical observations and data from animal models strongly point to persistent inflammation, activation of the complement 7 and coagulation pathways and to the damage of the alveolar integrity 8,9 as the most probable triggers of the fibrotic response. If not resolved, prolonged local inflammation will maintain uncontrolled healing responses, and produce excessive fibrous scar tissue within the lung.

Our group has developed and characterized a non-human primate model of sepsis induced ARDS that mimics the progression of the disease in humans 10. Using this model, we showed that sepsis ARDS in baboons is characterized by early, overlapping inflammatory and fibroproliferative phases that lead to increased collagen deposition, decrease in lung compliance and excessive fibrosis as detected by biochemical and histological biomarkers 10. We have also reported that complement activation show a bi-phasic time-course in response to sublethal *E. coli* challenge 11,12. The first wave of complement activation products occurs during the first 2–4 hrs during the bacteremia stage, while the second wave occurs after 8–24 hrs and it is likely induced by ischemia reperfusion injury 12. Moreover, we showed that treatment with compstatin, a C3 convertase complement inhibitor protects against *E. coli* induced organ failure in baboons 13.

As complement activation products could promote fibrosis in vital organs, like the lung 7, heart 14 and kidney 15, here we used gene expression approaches, coupled with biochemical and histological methods, to analyse the early protective effects of complement inhibition with compstatin on sepsis-induced fibrosis in the lung, focusing on multiple pathways involved in fibroblast biology and collagen deposition.

## Materials and methods

### Reagents

Antibodies and suppliers were as follows: rabbit monoclonal anti-human α-actin, proliferating cell nuclear antigen (PCNA), TIMP1, and vimentin (Epitomics, Burlingame, CA, USA); rabbit monoclonal anti-phospho-p44/42 MAP kinase (Thr202/tyr204) and phospho-Smad2 (Ser465/467)/Smad3 (Ser 423/425; Cell Signaling Technology, Danvers, MA, USA); rabbit polyclonal anti-human procollagen 3 (MD Biosciences, Zurich, Switzerland), β-actin, CTGF, and transforming growth factor-beta (TGF-β; Affinity Bioreagents, Golden, CO, USA); polyclonal goat anti-chemokine (C-X-C motif) ligand 12 CXCL12 and secreted protein acidic and rich in cysteine SPARC/osteonectin (R&D Systems, Minneapolis, MN, USA); mouse monoclonal anti-human hypoxia inducible factor (HIF1α) (Novus, Littleton, CO, USA), Rac1 (Abcam, Cambridge, MA, USA), Rho A (Santa Cruz Biotechnology, Santa Cruz, CA, USA); fluorescent or peroxidase-labelled secondary antibodies (Jackson ImmunoResearch, West Grove, PA, USA).

### Experimental procedures

The study was approved by the Institutional Animal Care and Use Committees of both Oklahoma Medical Research Foundation and the University of Oklahoma Health Science Center. The experimental procedure was as detailed previously 13. In brief, *Papio cyanocephalus* baboons were infused with 1 × 10^9^ live *E. coli* (LD50 dose). Compstatin analogue peptide (Ac-I[CVW(Me)QDWGAHRCT]I-NH_2_ was synthesized as described 16,17 and administered as a 10 mg/kg intravenous bolus, followed by 60 μg/kg/min. continuous infusion 13. The amount of compstatin used efficiently inhibited C3 activation during the infusion period 13.

The study comprised three experimental *E. coli* groups: (*i*) *E. coli* challenge only (*n* = 3); (*ii*) *E. coli* plus compstatin treatment from T0 to T+8 hrs (*n* = 4; prevention regimen, hereafter designated *E. coli* +CS T0); and (*iii*) *E. coli* plus compstatin from T+5 to T+11 hrs (*n* = 3; rescue regimen, *E. coli* +CS T+5). T0 treatment had targeted the pathogen-driven complement activation while the T+5 treatment aimed to inhibit the second wave occurring after bacteria clearance 12. The control group (three animals) received saline infusion only.

Physiological data and blood samples were collected and analysed as described 18.

Animals were killed at T+24 hrs, and lung tissue specimens were snap-frozen in liquid nitrogen and stored at −80°C or fixed for microscopy 19.

### Morphologic analysis

For immunofluorescence staining, tissues were fixed in 4% paraformaldehyde, washed with phosphate-buffered saline containing 15% sucrose, embedded in OCT, snap-frozen and stored at −80°C. Immunolabelling for procollagen-3, SPARC, phospho-MAPK, phospho-Smad, CTGF, PCNA, HIF1α, vimentin, α-actin, Rac1, RhoA and TIMP1 was performed as described 20. Cryosections (approximately 10-μm thick) were incubated with primary antibody (see ‘Reagents’) overnight at 4°C, followed by appropriate detection antibody coupled to FITC, then mounted with VectaShield hardset (Vector Labs, Burlingame, CA 94010, USA) supplemented with ToPro3 (Invitrogen, Carlsbad, CA, USA) as a nuclear counterstain. As a negative control for polyclonal antibody staining, the primary antibodies were replaced with an equivalent amount of rabbit nonimmune serum. mAb anti-digoxigenin (IgG1; Roche Diagnostics, Indianapolis, IN, USA), a hapten antigen that occurs only in plants, was used as isotype-matched control for mAb staining 20.

Immunofluorescence staining for procollagen-3, SPARC, phospho-MAPK, phospho-Smad, CTGF, PCNA, HIF1α, vimentin, α-actin, Rac1, RhoA and TIMP1 was performed as described 20. The samples were analysed by confocal laser scanning microscopy using a Nikon C1 scanning head mounted on a Nikon ECLIPSE 2000 U inverted microscope (Nikon Instruments Inc., Melville, NY, USA), equipped with either a 20× plan achromat objective (NA 0.46, dry) or a 60× apochromat objective (NA 1.2, water immersion).

The measurement of fluorescence intensity was carried out as described 20. In brief, 10–15 images (12-bit, 4095 grey levels/pixel) were collected for each experimental condition, and the mean fluorescence intensity (MFI) of the whole image or 15–20 regions of interest per image (as specified in the figure legends) was integrated using the EZ-C1 software (Nikon). Image collection parameters (neutral density filters, pinhole and detector gains) were kept constant during image acquisition to allow reliable comparisons between specimens. Images were analysed with ImageJ software (NIH, Bethesda, MD, USA).

Electron microscopy analysis of lung samples was performed as previously described 21. Lung samples were fixed in a mixture of 2% glutaraldehyde and 3% paraformaldehyde in cacodylate buffer, postfixed in 1% osmium tetraoxide and embedded in epoxy resin (Electron Microscopy Sciences, Fort Washington, PA, USA). Ultrathin sections (80 nm) were stained with uranyl acetate and lead citrate, and examined with a H-7600 transmission electron microscope (Hitachi, Tokyo, Japan).

### RNA isolation

Total RNA was isolated from frozen lung tissue using TRIzol reagent (Invitrogen, Life Technologies, Grand Island, NY, USA) according to manufacturer's protocol, then further purified with the RNeasy Tissue kit (Qiagen, Valencia, CA, USA), and the contaminant genomic DNA was removed with a Qiagen on-column DNase digestion kit. RNA concentration was measured with NanoDrop® ND1000 spectrophotometer (NanoDrop Technologies, Inc., Wilmington, DE, USA), and its integrity and purity were verified by Agilent 2100 Bioanalyzer Capillary Gel Electrophoresis System (Agilent, Palo Alto, CA, USA).

### Microarrays

Samples were processed according to Affymetrix recommendations, and cDNA was hybridized to the Affymetrix human U133A 2.0 array. The microarray data were analysed using Bioconductor tools 22 in R (version 2.7.2, http://www.r-project.org/). For normalization, a three-step robust multichip average method 23 in the Bioconductor package affy (http://www.bioconductor.org/packages/release/bioc/html/affy.html) was performed on the Affymetrix raw data (.cel files) to obtain log_2_ expression values for each probe set 24.

After filtering low expression-level probe sets, 8170 distinct genes resulted from averaging the measures of multiple probes mapped to the same gene. The data have been deposited in NCBI's Gene Expression Omnibus and are accessible through GEO Series accession number GSE23590 (http://www.ncbi.nlm.nih.gov/geo/query/acc.cgi?acc=GSE23590). Bioconductor package limma (http://www.bioconductor.org/packages/release/bioc/html/limma.html) 25 was used to find which probe sets showed significant differential expression under experimental conditions. A linear model was fitted to the expression data for each probe. Moderated *t*-statistics were computed by empirical Bayes shrinkage of the standard errors toward a common value. The *P*-values correspond to the moderated *t*-statistics. We used both *P*-values and fold change to determine a candidate probe list by requiring at least a twofold change (log2[fold]¦≥1) and *P* ≤ 0.05. Genes affected by *E. coli* were determined based on the ratio of healthy controls: *E. coli*. Genes affected by the compstatin treatment of septic baboons were determined based on the ratio *E. coli*+CST0:*E. coli* or *E. coli*+CS T+5:*E. coli*.

### Pathway analysis

Data sets of normalized expression values plus their associated gene identifiers were uploaded into IPA software (Ingenuity Systems, Mountain View, CA, USA; www.ingenuity.com) to generate biological networks, mapping values and gene identifiers (GenBank accession) to their corresponding gene objects in the Ingenuity Knowledge Base developed from published sources (Ingenuity Systems). In the current report, we focus exclusively on genes related to fibrosis, matrix deposition and remodelling.

### qPCR-based gene expression analysis

Real-time quantitative RT-PCR was used to determine the relative amount of mRNA transcripts in the baboon lung as described 13. Primers were designed using Primer Express software (Applied Biosystems, Life Technologies, Grand Island, NY, USA). Total RNA was extracted as described above. For each sample, 5 μg of total RNA was reverse-transcribed using the SuperScript III first-strand synthesis system for RT-PCR (Invitrogen) with random hexamer primers. qPCR was performed in duplicate with 2 μl of the 50-μl RT reaction products using iTaq SYBR Green Supermix with ROX kit (Bio-Rad, Hercules, CA, USA) in an ABI Prism 7000 sequence detection system (Applied Biosystems). Relative gene expression was estimated using ΔΔC_T_ method, following the manufacturer's protocol. The relative expression of target genes was normalized with β-actin or 18S rRNA.

### Protein extraction

Equal amounts of lung tissues were homogenized on ice with the extraction buffer provided in the antibody array kit (U.S. Biomax, Inc., Rockville, MD, USA). The extracts were centrifuged at 14,500 × g for 15 min., and the supernatants, representing the lung tissue lysates, were stored at −80°C. Total protein was determined using a BCA protein kit (Pierce, Rockford, IL, USA).

### Antibody array analysis

The expression profile of profibrotic signalling proteins was analysed with Jak/Stat Pathway Phospho and TGF-β signalling antibody arrays (U.S. Biomax, Inc.). Blocking, labelling, washing and fluorescence detection steps were performed according to the manufacturer's instructions. Slides were scanned with an Agilent DNA Microarray Scanner Model G2505B (Agilent Technologies, Inc., Santa Clara, CA, USA) and fluorescence intensity was measured by Imagene software (BioDiscovery, Marina del Ray, CA, USA).

### ELISA

Pro-collagen 3-N terminal peptide (P3-NP) in ethylenediaminetetraacetic acid plasma was measured with a kit from USCN Life Sci., Inc, Houston, TX, USA. DuoSet kits for human TIMP1 and CCL-2/monocyte chemotactic protein-1 (MCP-1) were from R&D Systems and the assays were performed according to manufacturer's instructions.

### Immunoblotting

Protein electrophoresis and transfer to polyvinylidene difluoride (PVDF) membranes were performed with the NuPAGE system (Invitrogen) and membranes were blotted with goat anti-CXCL12 polyclonal and anti-β-actin monoclonal antibodies. For detection, peroxidase-labelled antibodies and chemiluminescent substrate (ECL Plus; GE Healthcare, Little Chalfont, Buckinghamshire, UK) were used.

### Statistics

For statistical analyses, we used Prism (GraphPad Software, La Jolla, CA, USA). Values are given as mean ± S.E.M. The differences between *E. coli*–challenged groups, with/without compstatin treatment, were compared by a two-tailed, unpaired *t*-test, multiple *t*-test with Holm-Sidak multiple comparisons correction or one-way anova, followed by single comparison with the *E. coli*-challenged group using the Dunnett's test. Differences were considered significant when *P* < 0.05. All assays were performed at least in duplicate.

## Results

### Compstatin treatment decreases sepsis-induced expression of fibrosis genes in the lung

Using IPA, we identified fibrosis as a significantly altered canonical pathway during sepsis in baboons (Fig. S1). Compstatin treatment during the second stage of sepsis, beginning at 5 hrs post-exposure to *E. coli* (*E. coli* CS T+5) caused down-regulation of multiple fibrosis-related genes downstream of TGF-β, insulin-like growth factor 1 (IGF1), and ET1, including signalling mediators (Smad2/3, CTGF), matrix remodelling proteases/protease inhibitors (TIMP1), various collagens and other matrix proteins (fibronectin 1 (FN1), elastin (ELN), SPARC, versican (VCAN), syndecan 2(SDC2); Fig. S1).

**Figure 1:**
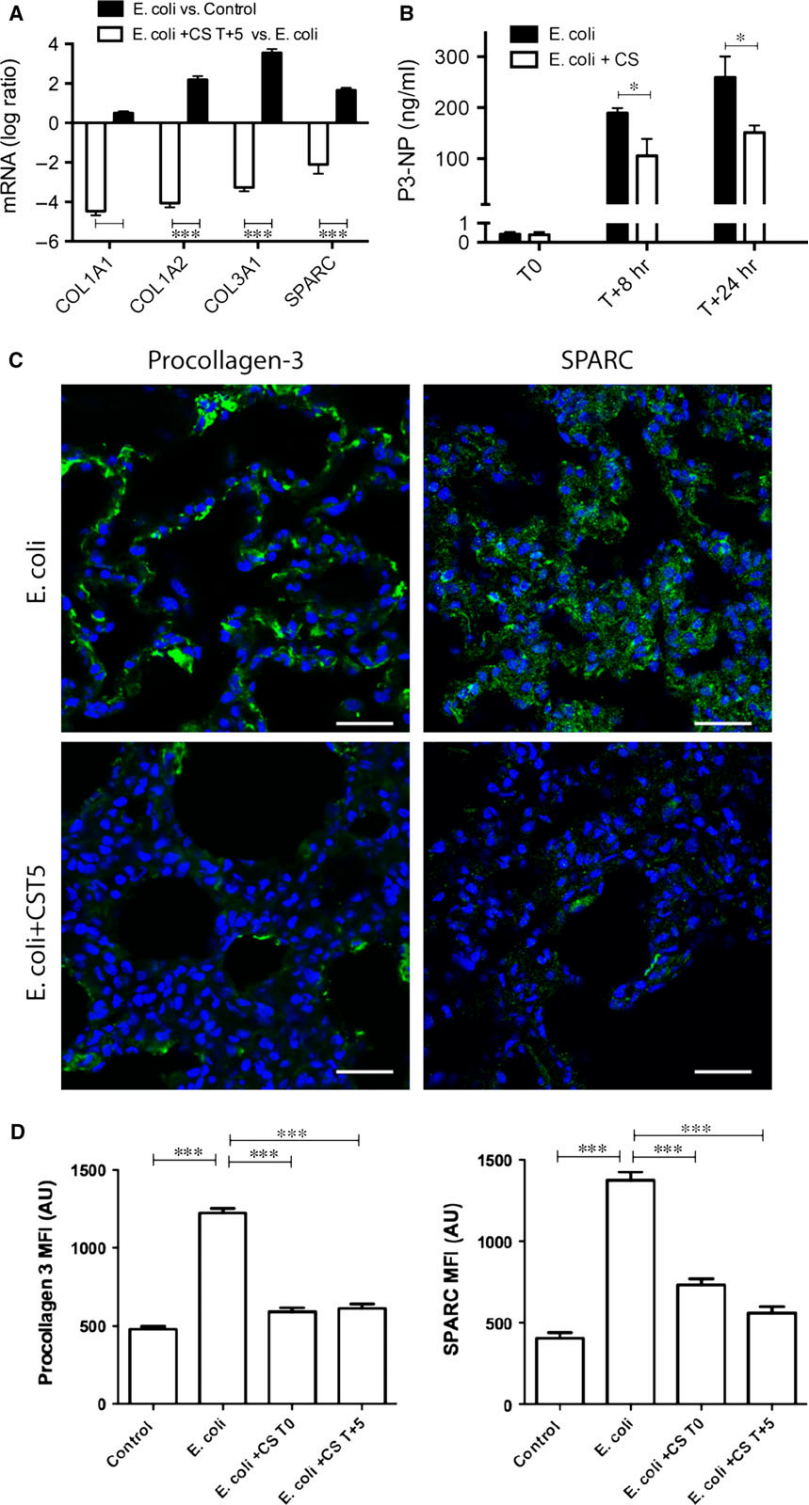
Compstatin treatment decreases sepsis-induced fibrosis of the lung. (A) Log ratio representation of qPCR analysis of three collagens and SPARC mRNA in sepsis *versus* healthy control and sepsis *versus* compstatin treated at T+5 hrs (*Escherichia coli* +CS T+5) animal groups. Expression values were normalized by housekeeping gene β-actin. Data are shown as mean ± S.E.M.; three replicates per experimental condition with unpaired two-tailed *t*-tests; ***P < 0.001. (B) Time-course of procollagen-3 N-terminal peptide (P3-NT) levels in plasma of baboons challenged with *E. coli*, with/without compstatin treatment at T+5. Data are shown as mean ± S.E.M.; *P < 0.05, *n* = 3. (C) Microscopic imaging and semi-quantitative analysis of staining intensity in lung tissue stained for matrix proteins and complement pathway proteins. The micrograph panel shows (in columns) immunostaining for procollagen 3 and SPARC (osteonectin) (in rows) in septic baboons (*E. coli*), and septic baboons treated with compstatin during the second (*E. coli* +CS T+5) stage of sepsis. Nuclear staining (blue) facilitates recognition of microscopic structures. Magnification: bar, 50 μm. (D) Histogram representation of mean fluorescence intensity (MFI) for procollagen-3 and SPARC. In addition to the image series shown above, all quantitative image analysis includes data obtained from healthy controls and compstatin treatment during the first stage of sepsis (*E. coli* +CS T0). Data are shown as means ± S.E.M. of at least 10 images for each experiment. One-way anova with Dunnett's multicomparison test; ***P < 0.001 as compared to the *E. coli* group.

qPCR confirmed the microarray results showing downregulation of three collagens (COL1A1, COL1A2 and COL3A1) and SPARC (osteonectin) transcripts (Fig.1A). Plasma P3-NP levels, a marker of active collagen 3 synthesis, decreased significantly in compstatin-treated septic animals as compared to non-treated group (Fig.1B). Both sepsis-induced matrix production and the downregulating effect of compstatin treatment were confirmed by semi-quantitative histochemical analysis after immunostaining for procollagen-3 and SPARC (Fig.1C and D).

### Compstatin treatment inhibits pro-fibrotic signalling pathways, as well as hypoxic, angiogenic and stress response genes activated by sepsis

Functional IPA showed that *E. coli* sepsis strongly induced genes of the complement system and multiple growth factor-controlled signalling pathways, including insulin growth factor (IGF1), epidermal growth factor (EGF), platelet-derived growth factor, hepatocyte growth factor (HGF), fibroblast growth factor (FGF) and TGF-β signalling, as well as pathways involved in myofibroblast differentiation, proliferation and matrix production (renin-angiotensin, endothelin and chemokine signalling; Fig. S2A). Inhibition of complement with compstatin (*E. coli* CS T+5) effectively decreased the expression of all these gene classes (Fig. S2B).

*Escherichia coli* sepsis increased the hypoxic response in the lung, causing a 2.4-fold increase in HIF1α transcripts (Fig. S3A), strong immunofluorescent staining for HIF1α (Fig. S3B), and up-regulation of two downstream HIF1α-controlled genes (SERPINE1, 3.8-fold; lactate dehydrogenase A (LDHA), 3.5-fold). Treatment with compstatin counteracted these effects by decreasing both HIF1α transcription (2.2-fold *versus* infected baboons not receiving compstatin) and the protein immunostaining, as well as the expression of other hypoxia-related transcription factors and co-activators (EP300, −2.5; CREB1, −1.75; SUMO, −1.7) and hypoxia-regulated genes involved in angiogenesis and fibrosis (VEGF, −2.39; CYR61, −4.06; ET1, −2.37, LDHA, −1.18) (Fig. S3A and C).

In addition, compstatin treatment decreased stress response proteins that were up-regulated by sepsis-induced thermal, proteotoxic and oxidative stress, including heat shock proteins (HSP; Fig. S4A) and protein disulfide isomerases (Fig. S4B). The decrease in HSP expression correlated with lower expression of several nuclear pore proteins and lamins (Fig. S4C) that are part of nuclear complexes controlling transcription activation after heat shock 26.

### Complement inhibition decreases TGF-β signalling and the expression of TGF-β-driven genes

**Figure 2:**
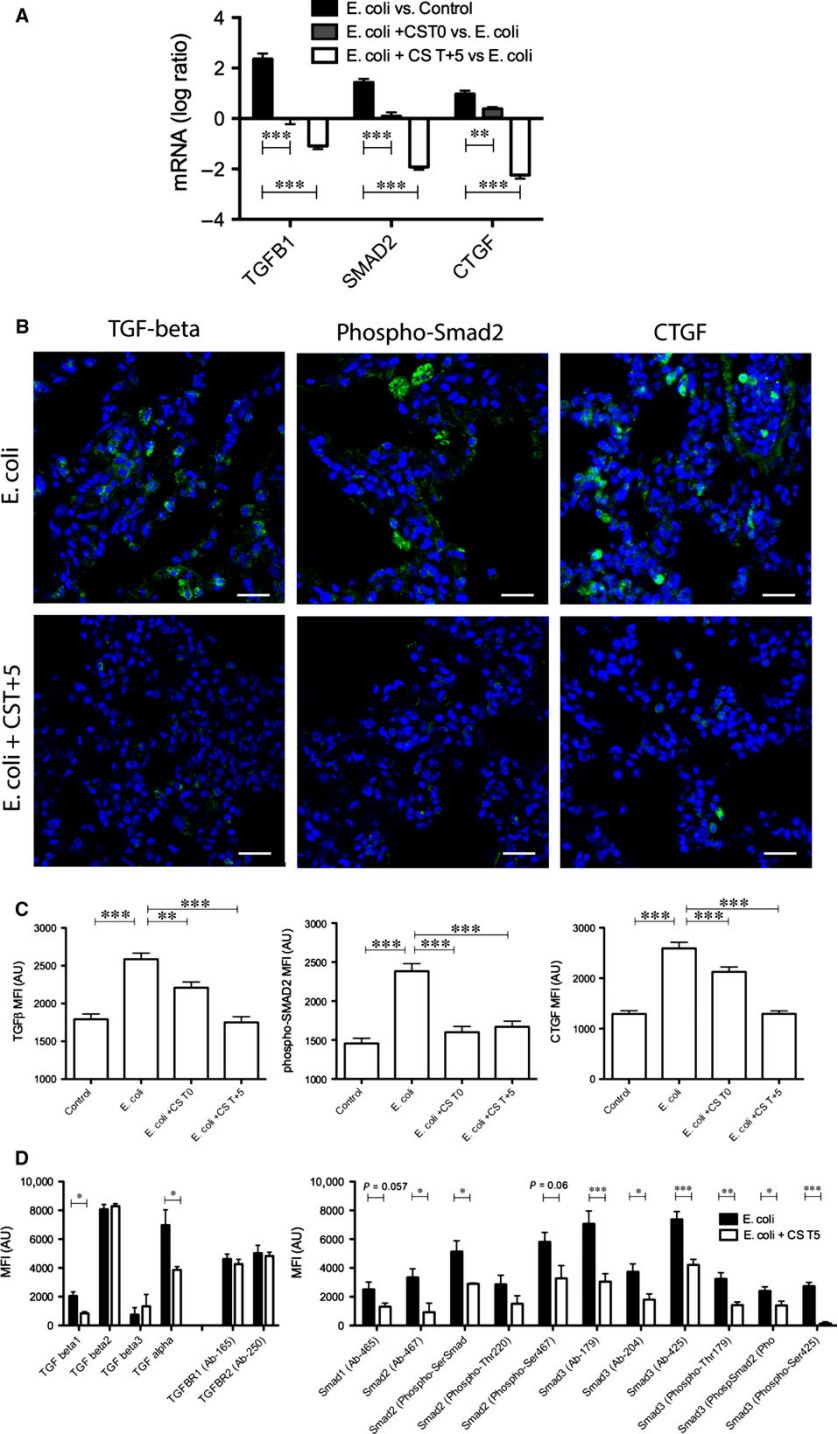
Immunolocalization and semi-quantitative analysis of fluorescence intensity in lung tissue stained for proteins contributing to fibrosis *via* transforming growth factor-beta (TGF-β) signalling. (A) Log ratio representation of qPCR analysis of TGF-B1, SMAD2 and CTGF transcripts in *Escherichia coli versus* healthy controls and animals treated with compstatin at T0 or T+5, *versus* untreated septic animals. Expression values were normalized by housekeeping gene β-actin. Data are shown as mean ± S.E.M., *n* = 3; one-way anova with Dunnett's multicomparison test. **P < 0.01, ***P < 0.001 as compared to *E. coli versus* Control ratio. (B) Immunostaining for TGF-β, phospho-Smad2/Smad3, and CTGF (columns) in the lungs of septic baboons (*E. coli*) *versus* compstatin-treated baboons at T+5 (*E. coli* + CS T+5) (rows). Magnification: bars, 50 μm. (C) Histogram representations of mean fluorescence intensity (MFI) of images collected for the above-mentioned proteins as detailed in Figure1. Data are shown as means ± S.E.M.; one-way anova with Dunnett's multicomparison test. ***P < 0.001 as compared to the *E. coli* group. (D) Phospho-antibody array analysis. MFI of the antibody spots are presented as means ± S.E.M. of six replicates per experimental condition with unpaired two-tailed *t*-tests. *P < 0.05; **P < 0.01; ***P < 0.001.

As our pathway analysis (Fig. S1) revealed that compstatin treatment significantly affected TGF-β signalling, we performed qPCR, immunohistochemistry and antibody array techniques to characterize the mRNA and protein expression of TGF-β and two key proteins involved in this pathway. Detection of the phosphorylated Smad2 and CTGF by immunostaining and/or antibody array served as markers for TGF-β signalling activity. *Escherichia coli* sepsis significantly increased the TGF-β, Smad2 and CTGF transcripts (Fig.2A), and enhanced their staining intensity (Fig.2B and C) on tissue sections, as well as their amounts in lung homogenates (Fig.2D). Compstatin treatment consistently decreased the mRNA and protein expression of all three proteins (Fig.2A–D).

### Compstatin treatment reduces myofibroblast differentiation and cell migration and proliferation

The functional IPA revealed that *E. coli* sepsis strongly modulates the expression of genes involved in connective tissue production and fibroblast transformation as well as chemotaxis and cell migration (Table1, Figs S5 and S6), while compstatin treatment of septic baboons at T+5 significantly dampened these effects.

**Table 1:** Pathways that are significantly modified by *Escherichia coli* sepsis *versus* healthy controls and by compstatin treatment at T+5 *versus E. coli* challenge only

	*E. coli*				*E. coli* + CS T+5			
	Modified genes			*P*-value	Modified genes			*P*-value
	Up	Down	Total		Up	Down	Total	
Fibroblast transformation	32	10	42	1.41E-06	12	69	81	1.09E-07
Connective tissue disorder	133	59	192	2.62E-05	92	341	434	3.1E-07
Chemotaxis	39	13	52	5.0E-03	31	92	123	1.98E-05
Cell migration	95	40	135	2.11E-05	80	250	330	9.36E-08
Cell proliferation	187	70	257	2.67E-08	118	466	584	5.83E-13
Fibroblast proliferation	23	7	30	4.80E-03	10	62	72	5.56E-06

**Figure 3:**
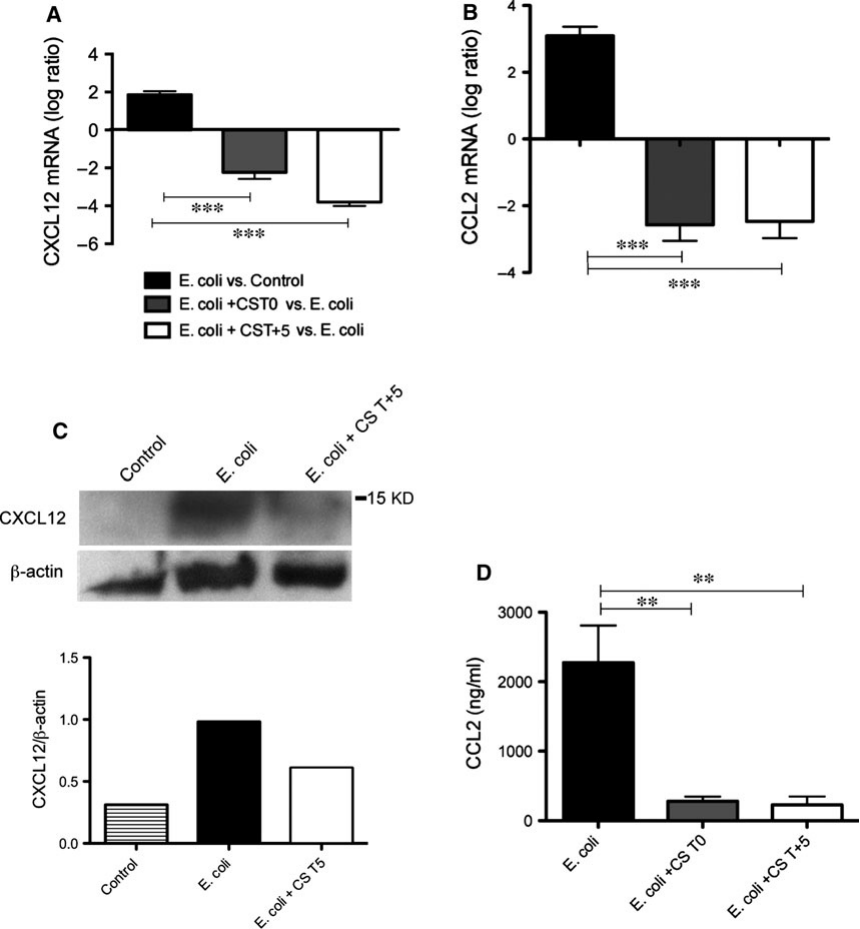
Compstatin treatment decreases sepsis-induced fibrogenic cytokines. (A) Log ratio representation of qPCR analysis of CXCL12 mRNA transcripts in *Escherichia coli versus* healthy controls and animals treated with compstatin at T0 or T+5, *versus* untreated septic animals. Data are shown as mean ± S.E.M. of three replicates. One-way anova with Dunnett's multicomparison test; ***P < 0.001 as compared to *E. coli versus* Control ratio. (B) CCL2 mRNA levels, determined by qPCR. Data are shown as mean ± S.E.M. of three replicates. One-way anova with Dunnett's multicomparison test; ***P < 0.001 as compared to *E. coli versus* Control ratio. (C) Immunoblot and densitometry of CXCL12 in lung homogenates from control, septic (*E. coli*), and compstatin-treated (*E. coli* +CS T+5) baboons. β-actin was used for normalization. Representative data of three replicates are shown. (D) CCL2 levels, determined by ELISA in plasma collected 24 hrs post-challenge from *E. coli* sepsis and compstatin treatment groups. Data are shown as mean ± S.E.M. of three replicates. One-way anova with Dunnett's multicomparison test; **P < 0.01 as compared to *E. coli* group.

Remarkably, compstatin treatment decreased the mRNA expression of profibrotic chemokine genes induced by *E. coli* sepsis, such as CXCL12 (stromal cell-derived factor 1 (SDF-1); Fig.3A), which controls the post-injury recruitment of circulating fibrocytes into organs 27, and CCL2 (MCP1), which promotes fibroblast survival 28 (Fig.3B). Immunoblotting of lung homogenates revealed that CXCL12 was strongly induced by sepsis and decreased by compstatin (Fig.3C). ELISA of tissue homogenates confirmed the decrease in CCL2 in both T0 and T+5 treatment groups (Fig.3D).

**Figure 4:**
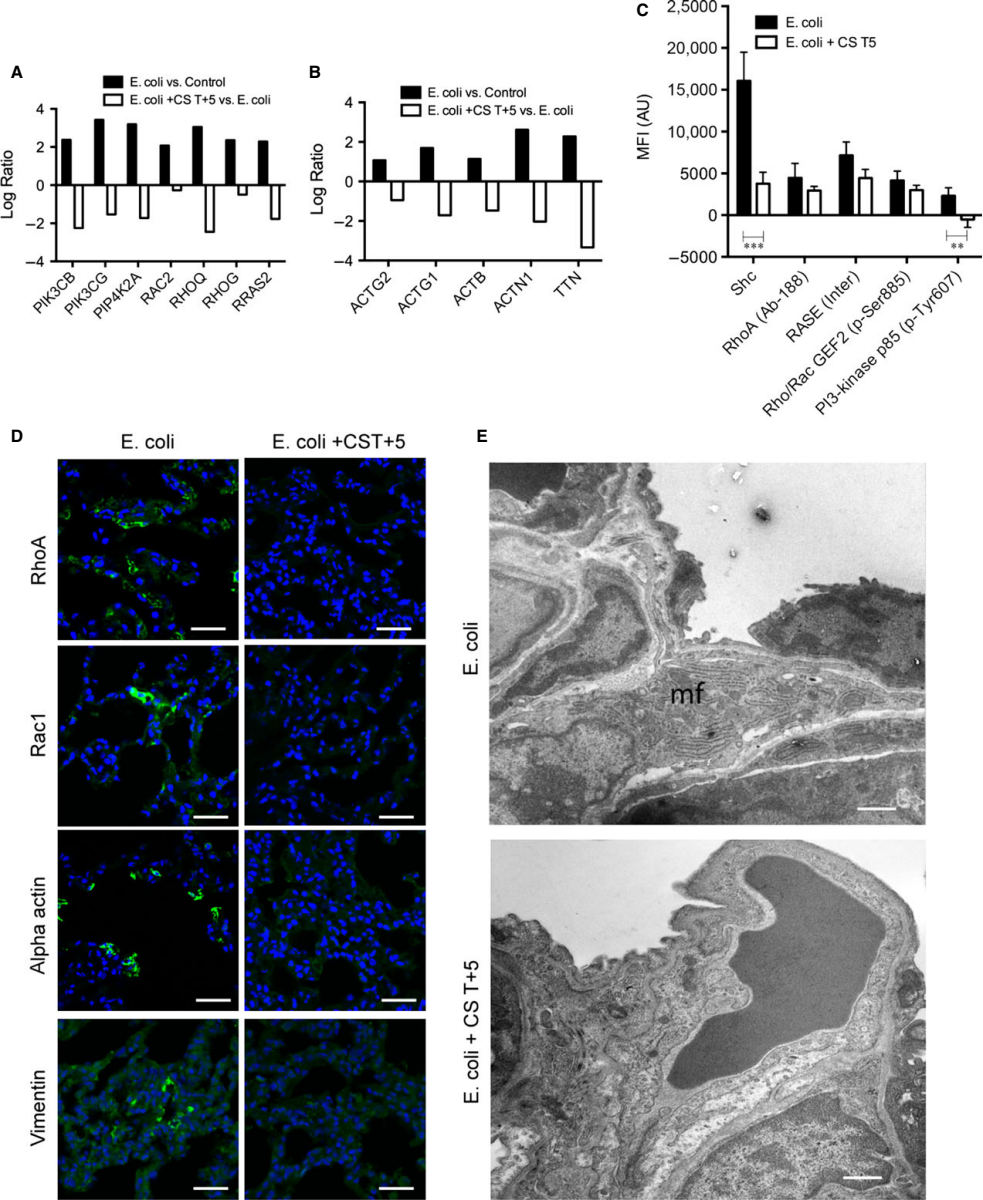
Compstatin treatment inhibits sepsis-induced myofibroblast differentiation and contractility. (A and B) Log ratio of microarray data showing expression of molecules involved in Rho/Rac and PI3K signalling (A) and actins and actin-binding proteins (B). Data are shown as log ratio mean of three biological replicates. (C) Phospho-antibody array analysis showing expression and phosphorylation of molecules involved in Rho/Rac and PI3K signalling. Mean fluorescence intensity (MFI) of the antibody spots are presented as mean ± S.E.M. of six replicates per experimental condition with unpaired two-tailed *t*-test. **P < 0.01; ***P < 0.001. (D) Immunocytochemical staining for detection of vimentin and α-actin-positive cells and for cell migration-relevant signalling molecules Rac and Rho in sepsis (*Escherichia coli*) *versus* compstatin-treated (*E. coli* + CS T+5) animals. Magnification: bars, 50 μm. (E) Transmission electron micrographs demonstrate the presence of cells with ultrastructural characteristics of a secretory myofibroblast (mf) within the alveolar walls of animals from sepsis (*E. coli*) but not compstatin-treated (*E. coli* + CS T+5) baboons. Magnification: bar, 1 μm.

Compstatin treatment significantly downregulated multiple genes participating in G-protein-coupled receptors (GPCR-) and integrin-dependent actin polymerization *via* the Rho and Rac pathways, which control fibroblast migration and spreading (Fig.4A). Accordingly, we observed decreased mRNA levels for RHOQ and RHOG (Rho GTPases), PIK3CB and PIK3CG, actins, myosins, actin-binding proteins, titin, and the phosphatidylinositol kinases PI4KA and PIP4KA2 (Fig.4B). Antibody array analysis confirmed that compstatin downregulated Rac/Rho/PI3K signalling proteins, like Shc, phospho-PI3K p85-α, RhoA, and Ras/p21 (Fig.4C). Also, lung samples from septic animals stained positively for two myofibroblast markers, vimentin and α-actin, as well as Rac1 and Rho1; samples from compstatin-treated animals showed considerably lower levels of staining for these proteins, suggesting reduced fibroblast infiltration (Fig.4D). Similarly, electron microscopy frequently showed myofibroblasts with hypersynthetic and contractile phenotypes in the lungs of *E. coli*-challenged, but not compstatin-treated, septic baboons (Fig.4E).

**Figure 5:**
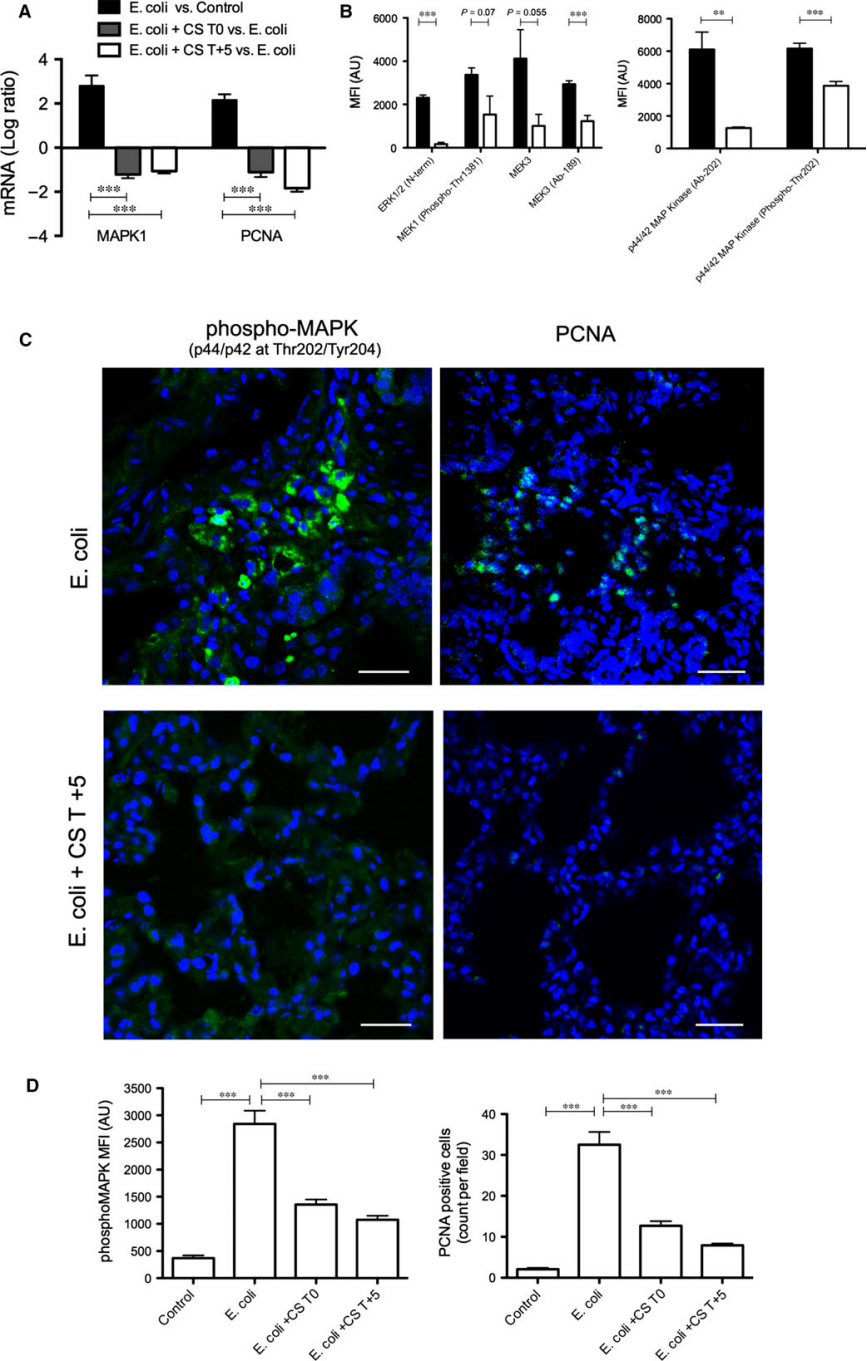
Compstatin inhibits sepsis-induced myofibroblast proliferation. (A) Log ratio of qPCR data showing transcript levels for proliferation signalling (MAPK1) and marker (PCNA) proteins in *Escherichia coli* + CS T+5 *versus E. coli* animal groups. Expression values were normalized by housekeeping gene β-actin. Data are shown as mean ± S.E.M. of three replicates. One-way anova with Dunnett's multicomparison test; ***P < 0.001 as compared to *E. coli versus* Control ratio. (B) Phospho-antibody array analysis showing expression and phosphorylation of molecules involved in ERK and MEK proliferation signalling. Mean fluorescence intensity (MFI) data for antibody spots are presented as mean ± S.E.M. of six replicates per experimental condition with unpaired two-tailed *t*-test. **P < 0.01; ***P < 0.001. (C) Immunofluorescence staining for phospho-MAPK1 and PCNA in foci in the lungs of septic baboons, but not in compstatin-treated animals. Magnification: bar, 50 μm. (D) Histogram representation of MFI of images collected for the above-mentioned proteins. Data are shown as means ± S.E.M.; one-way anova with Dunnett's multicomparison test. ***P < 0.001 as compared to the *E. coli* group.

Compstatin also downregulated genes related to cell proliferation that have been induced by *E. coli* sepsis (Table1; Fig. S5). qPCR analysis showed that two reliable markers of cell proliferation, MAPK1 and PCNA, were up-regulated by sepsis and decreased by compstatin treatment both at T0 and T+5 (Fig.5A). Antibody array analysis showed concomitantly decreased total amounts and phosphorylation levels of proteins in the MAPK signalling pathway, including ERK1/2, phospho-MEK1, and MAPK1 (Fig.5B) in the compstatin-treated *versus* non-treated septic baboons. Semi-quantitative analysis after immunostaining for the activated form of MAPK1 (phospho-Thr202/Tyr204 MAPK1), a master regulator of cell proliferation, and for PCNA, which is expressed only by proliferating cells, showed significantly increased fluorescence intensity in foci of proliferating cells in the lung of septic, but not compstatin-treated, animals (Fig.5C and D).

### Compstatin treatment inhibits sepsis-induced extracellular matrix remodelling

**Figure 6:**
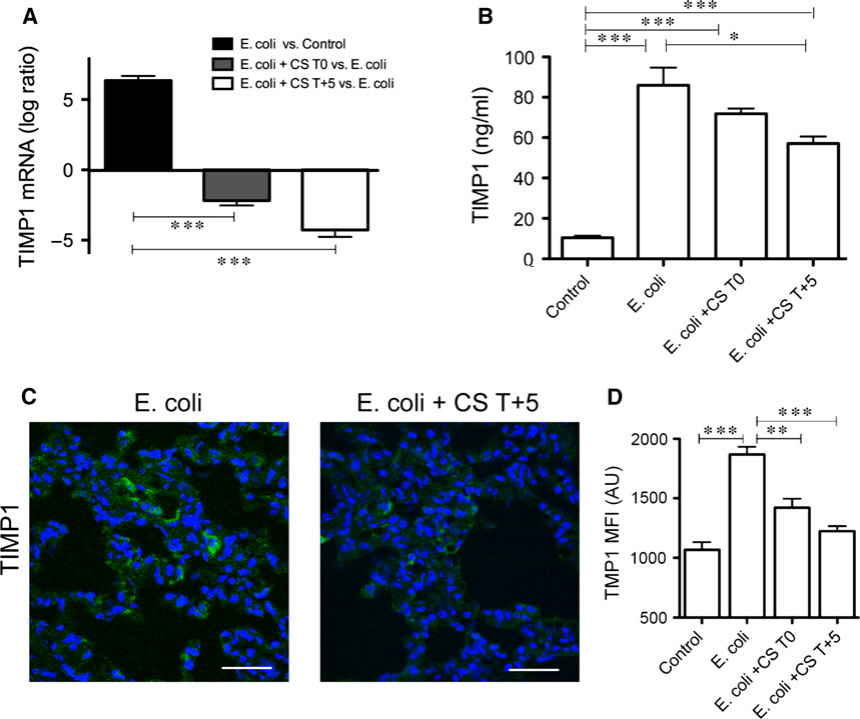
Compstatin inhibits sepsis-induced expression of TIMP1. (A) Log ratio qPCR data showing transcript regulation of TIMP1 by sepsis and complement inhibition. Expression values were normalized by housekeeping gene β-actin. Data are shown as mean ± S.E.M. of three replicates. One-way anova with Dunnett's multicomparison test; ***P < 0.001 as compared to *Escherichia coli versus* Control ratio. (B) TIMP1 levels in citrated plasma at 24 hrs after challenge with *E. coli* with/without compstatin treatment during the first (T0) and second (T+5) stages of experimental sepsis. Data are shown as mean ± S.E.M. of three replicates. One-way anova with Dunnett's multicomparison test; ***P < 0.001, *P < 0.05 as compared to Control or *E. coli* groups. (C) Immunofluorescent staining and semi-quantitative image analysis (D) confirm the induction of TIMP1 by sepsis and the inhibitory effect of compstatin on this protease inhibitor. Magnification: bar, 50 μm. Data are shown as means ± S.E.M.; one-way anova with Dunnett's multicomparison test. ***P < 0.001, **P < 0.01, as compared to the *E. coli* group.

As changes in the turnover of the extracellular matrix could contribute to fibrosis, we also examined the expression of extracellular proteases. Sepsis induced the expression of three members of the disintegrin and metalloproteinase (ADAM) family, ADAM17, ADAM19 and ADAM10, and compstatin treatment mitigated the enhancement of these genes (Fig. S7). Moreover, compstatin counteracted the effects of *E. coli* sepsis on the expression of matrix metalloproteinases MMP8, MMP9 and MMP14 (Fig. S7). The most prominently induced was tissue MMP inhibitor 1 (TIMP1), which was 6.34-fold increased in sepsis and 4.52-fold decreased in compstatin-treated septic baboons, as shown by qPCR (Fig.6A). TIMP1 levels in plasma at 24 hrs post-challenge (Fig.6B) correlated well with TIMP1 mRNA. Similarly, semi-quantitative immunofluorescence analysis (Fig.6B and C) of lung tissues stained for TIMP1 supports the mRNA and protein quantification, with specimens from the sepsis group being stronger stained for TIMP1, while compstatin-treated baboons showed significantly lower staining. Moreover, sepsis induced, and compstatin counteracted, the expression of the tissue plasminogen activator (PLAT) and six acute-phase reactant serpins, including SERPINE1 (PAI-1, the main plasma inhibitor of plasminogen activation; Fig. S8), which confirmed our previous protein quantification 13.

## Discussion

In this study, we used transcriptomics, phospho-proteomics, biochemical, and immunohistochemical analyses to characterize the effect of a potent C3 convertase inhibitor, compstatin, on fibrosis-specific pathways in the lung of baboons exposed to severe sepsis. We focused our investigation on early fibrotic events occurring at 24 hrs post-challenge for the following reasons: (*i*) we have shown previously that in our model, this time-point is dominated by robust induction of genes involved in synthesis of extracellular matrix and tissue remodelling 21; (*ii*) our previously reported detailed time-course analysis of the pathophysiology of sepsis ARDS in baboons demonstrated that animals surviving experimental severe sepsis could develop structurally and functionally detectable lung fibrosis 10; (*iii*) elevation of fibrosis biomarkers, such as procollagen N-terminal peptides could already be detected within 24 hrs after acute lung injury in the plasma of human patients 3,6; (*iv*) plasma levels of an established biomarker of active fibrogenesis, P3-NP peaked at 24 hrs post-challenge in our baboon model of *E. coli* sepsis (Fig. S9); and (*v*) early interventions during sepsis progression are critical to prevent organ damage 29, therefore inhibition of matrix deposition by a complement inhibitor has the potential to prevent the long-term debilitating effects of sepsis-induced lung scarring. Here, we demonstrate that complement inhibition effectively attenuates the early profibrogenic responses in the lung of *E. coli* challenged baboons, including fibroblast differentiation, cell migration and proliferation, and the enhanced production of collagens and other matrix proteins.

*Escherichia coli* sepsis in baboons exhibits two distinct stages of disease progression 11. The first stage is a highly coagulopathic, inflammatory response to the bacteria, which leads to intravascular fibrin deposition and hypoperfusion of vital organs. Ischemia up-regulates hypoxia-inducible genes, increases neutrophil adherence and transmigration and leads to oxidative burst with enhanced production of reactive oxygen and nitrogen species 11. These events fuel the second stage by promoting tissue injury and complement activation, which predispose to organ dysfunction and may lead to organ failure and death 11.

We have previously shown that baboons that survive severe sepsis develop chronic inflammation and fibrosis of the lung in the next 6–27 months following the acute sepsis episode 10. *Escherichia coli* sepsis induces deposition of complement activation products in vital organs, including the lung and complement inhibition with compstatin attenuates inflammation and hemostatic dysfunction, restores systemic blood pressure, and improves organ function during *E. coli* sepsis in baboons, even when was given during the second stage of progressive organ failure 13.

Our results demonstrate a robust expression of profibrogenic and matrix remodelling genes as early as 24 hrs post-*E. coli* challenge. This response is likely driven by the increased endothelial and epithelial permeability and subsequent capillary leakage and intra-alveolar oedema that occur during the first stage, as well as by the later host responses driven by oxidative stress 10,11,21.

Acute injury of the lung up-regulates fibrosis-specific pathways, like TGF-β, IGF1 and ET1, which increase the recruitment and differentiation of myofibroblasts, cell proliferation and production of extracellular matrix 2,10. As we have previously shown 10, foci of proliferating myofibroblasts that appear after the first day post-challenge may initiate a robust focal fibrotic process. This is consistent with clinical studies that showed elevation of profibrotic markers within 24 hrs of acute lung injury, suggesting that fibrosing alveolitis begins early in the course of the disease 6. Moreover, high procollagen-3 levels in the alveolar fluid correlate with poor prognosis, suggesting that fibrosis is a marker of severe lung injury 3.

Compstatin treatment consistently reduced the expression of fibrosis-related genes and concomitantly prevented the accumulation of fibroblasts and production of matrix proteins at 24 hrs post-sepsis challenge, as shown by decreased immunostaining for procollagens 1 and 3 and lower plasma levels of P3-NP in compstatin-treated *versus* non-treated septic baboons. Procollagens and peptides released during collagen fibre formation are reliable fibrosis markers reflecting the rate of collagen synthesis and extracellular deposition in septic patients 3,6,30. The turnover of lung collagen is strongly increased during the first 24 hrs after diagnosis 3,6, particularly in non-survivors 4, and increased collagen mRNA was observed immediately after lung damage 31. The content in collagen of the lung is more than double in patients who survive longer than 2 weeks post-ARDS 5. Early increase in collagen synthesis indicates the degree of acute organ dysfunction/failure and represents a biomarker of risk stratification of patients with sepsis 32.

Altogether, our data corroborate with clinical observations and strongly support the conclusion that profibrotic events are triggered in the lung during acute sepsis and that complement activation supports this process, as also suggested by mouse and human studies. Extensive complement activation is detected in the blood and bronchoalveolar fluid of patients with interstitial pneumonitis 33, indicating potential association of complement activation with subsequent pulmonary fibrosis. Complement depletion attenuates bleomycin-induced fibrosis in mice 34. The fibrogenic effect of complement activation in mice seems to occur upstream of C5, as up-regulation of TGF-β does not occur until the later stages of bleomycin-induced pulmonary fibrosis in C5-deficient mice 7. Complement activation products also represent risk factors for fibrosis of the liver 35 and kidney 36.

Our data show that the anti-fibrotic effects of compstatin occur at multiple levels. *Escherichia coli* sepsis is a powerful inducer of tissue factor (TF)-mediated coagulation 19. Fibrin formation coupled with impaired fibrinolytic activity resulting from PAI1 up-regulation contributes to persistent intra-alveolar fibrin clots. Fibrin itself acts as a conduit for fibroblast migration into the alveolar wall and air spaces and allows focal fibroblast proliferation, thus contributing to the fibrotic remodelling process. As complement inhibition reduced the thrombogenic response in septic baboons by inhibiting TF and PAI-1 expression 13, it is likely that the shift in the coagulant/anticoagulant balance towards anticoagulation or fibrinolysis induced by complement inhibitors could attenuate fibrogenic reactions in the lung.

We have previously shown that sepsis leads to inflammation 10, and compstatin treatment reduced the number of macrophages accumulating in the lung by 24 hrs post-challenge 13. During the late stage of sepsis, macrophages differentiate into the M2 phenotype that features, among other cytokines, large amounts of TGF-β 10, a master regulator of fibrosis (reviewed in Ref. 8). Sepsis strongly increased Smad2/3 phosphorylation 10, a marker of TGF-β signalling, but compstatin treatment significantly reduced this effect. Thus, downregulation of TGF-β signalling could mediate the antifibrotic effect of complement inhibition.

We also observed that compstatin treatment efficiently reduced the expression of sepsis-induced chemokines involved in fibrocyte recruitment into tissues, including CCL2 (MCP1) and CXCL12. CCL2 is markedly elevated in patients with pulmonary fibrosis 37, and a reduction in CCL2 in bleomycin-induced lung injury attenuates collagen deposition 38.

Compstatin treatment downregulated multiple sepsis-induced regulators of extracellular matrix remodelling; TIMP1 decreased by >fourfold in the compstatin-treated group. This protein inhibits the activity of all known MMPs, and its expression is regulated by C5a-dependent signalling 35. TIMP1 could enhance fibrosis by inhibiting MMP-dependent matrix degradation. High TIMP1 levels associate with extensive fibrosis in patients and animal models 39, and predict poor clinical outcome in septic patients 40. Two major ‘sheddases’, ADAM17 and ADAM10, which have strong proinflammatory and profibrotic effects are also decreased by compstatin treatment. ADAM17 and ADAM10 promote endothelial permeability and fibroblast migration by cleaving cell adhesion molecules, and stimulate fibroblast proliferation by releasing and activating ligands of TGF and EGF receptors 41.

Taken together, these data suggest that in our baboon model, complement inhibition acting directly on fibrosis-specific pathways and indirectly by reducing lung injury and inflammation protects against collagen deposition in the lung, a sequel of sepsis-induced acute lung injury. This conclusion comes with two caveats. First, the model may not reflect the whole diversity of sepsis, as it rather mimics the fulminant infections with Gram-negative bacteria, which are typically accompanied by massive complement activation and disseminated intravascular coagulation. Secondly, a longer term follow-up of compstatin-treated animals has not been performed so far, therefore further studies are warranted to validate these results. Patients with severe sepsis experience ongoing morbidity as a result of non-resolved inflammation and fibrosis, which extend far beyond the 28-day end-point of most clinical trials, decrease life expectancy and impair long-term quality of life for sepsis survivors 42. As mortality from sepsis continue to decline because of better supportive therapies 43, it becomes increasingly important to attenuate sepsis-related long-term morbidity. Because the fibrotic response is initiated during the early stages of sepsis, it is plausible that complement inhibition during the acute phase of the disease could protect against long-term pulmonary morbidity in sepsis survivors.

## Clinical relevance

Acute respiratory distress syndrome is followed by an early fibroproliferative reaction that contributes to morbidity and mortality in sepsis survivors. We demonstrate that complement inhibition efficiently attenuates the fibrotic response by decreasing TGF-β signalling, fibroblast differentiation, migration, and proliferation, and extracellular matrix remodelling.

## Supporting Information

**Figure S1** Compstatin treatment prevents sepsis-induced up-regulation of matrix proteins in the lung. **Figure S2** Canonical pathway up-regulated by sepsis (A) and down-regulated by compstatin treatment of septic baboons. **Figure S3** Compstatin treatment down-regulates hypoxia inducible genes. **Figure S4** Compstatin treatment down-regulates sepsis induced hypoxia. **Figure S5** Expression of cellular stress response genes. **Figure S6** Lists of pathway-specific differential expressed genes involved in cell proliferation. **Figure S7** Lists of pathway-specific differential expressed genes involved in migration/chemotaxis. **Figure S8** Expression of genes involved in extracellular matrix remodelling. **Figure S9** Time course of procollagen 3 N-terminal peptide levels in plasma of baboons challenged with LD50 *E. coli*.
