# Supplementary material for: Complement inhibition decreases early fibrogenic events in the lung of septic baboons
Source: J Cell Mol Med. 2015 Sep 3;19(11):2549–63. doi: 10.1111/jcmm.12667 (PMC4627561; doi:10.1111/jcmm.12667)
Supplement: Supplementary file 1 — Figure S1 Compstatin treatment prevents sepsis-induced up-regulation of matrix proteins in the lung. Figure S2 Canonical pathway up-regulated by sepsis (A) and down-regulated by compstatin treatment of septic baboons. Figure S3 Compstatin treatment down-regulates hypoxia inducible genes. Figure S4 Compstatin treatment down-regulates sepsis induced hypoxia. Figure S5 Expression of cellular stress response genes. Figure S6 Lists of pathway-specific differential expressed genes involved in cell proliferation. Figure S7 Lists of pathway-specific differential expressed genes involved in migration/chemotaxis. Figure S8 Expression of genes involved in extracellular matrix remodelling. Figure S9 Time course of procollagen 3 N-terminal peptide levels in plasma of baboons challenged with LD50 E. coli. [file jcmm0019-2549-sd1.pdf]

A

B

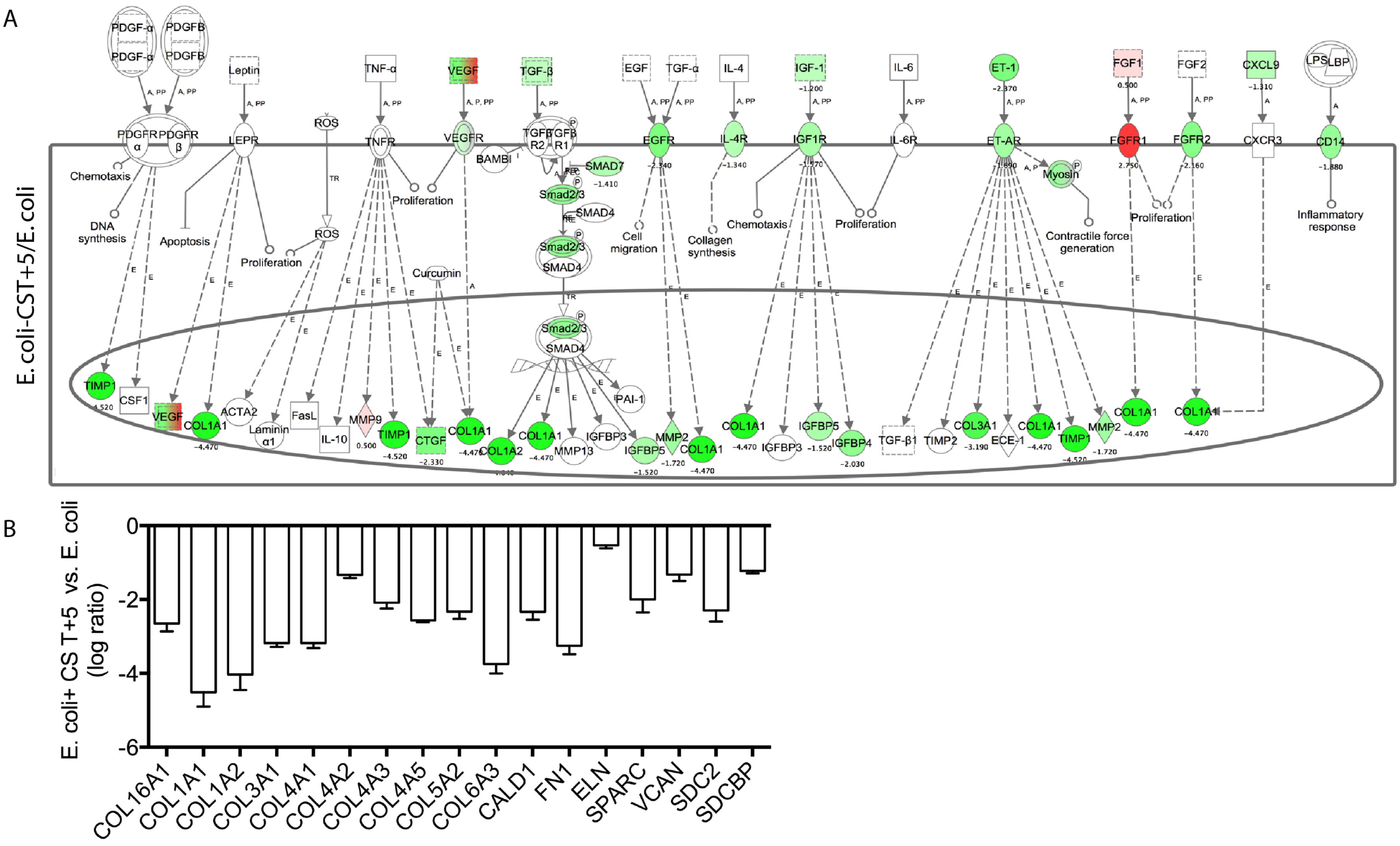

Figure E1. Compstatin treatment prevents sepsis-induced upregulation of matrix proteins in the lung.
(A): IPA canonical pathway analysis reveals that compstatin (CS) treatment during the second stage of E. coli sepsis (E. coli +CS T + 5) leads to downregulation (green) of several sepsis-induced signaling pathways involved in fibrosis, including TGF-β, EGF, IGF, endothelin-1 (ET1) and FGF. (B): Log ratio representation of multiple extracellular matrix genes, including 10 collagens, fibronectin (FN1), elastin (ELN), versican (VCAN), syndecan 2 (SDC2) and syndecan binding protein, all downregulated by compstatin during the second stage of sepsis (E. coli +CS T + 5). Data are shown as log ratio mean ± SEM of 3 biological replicates.

A

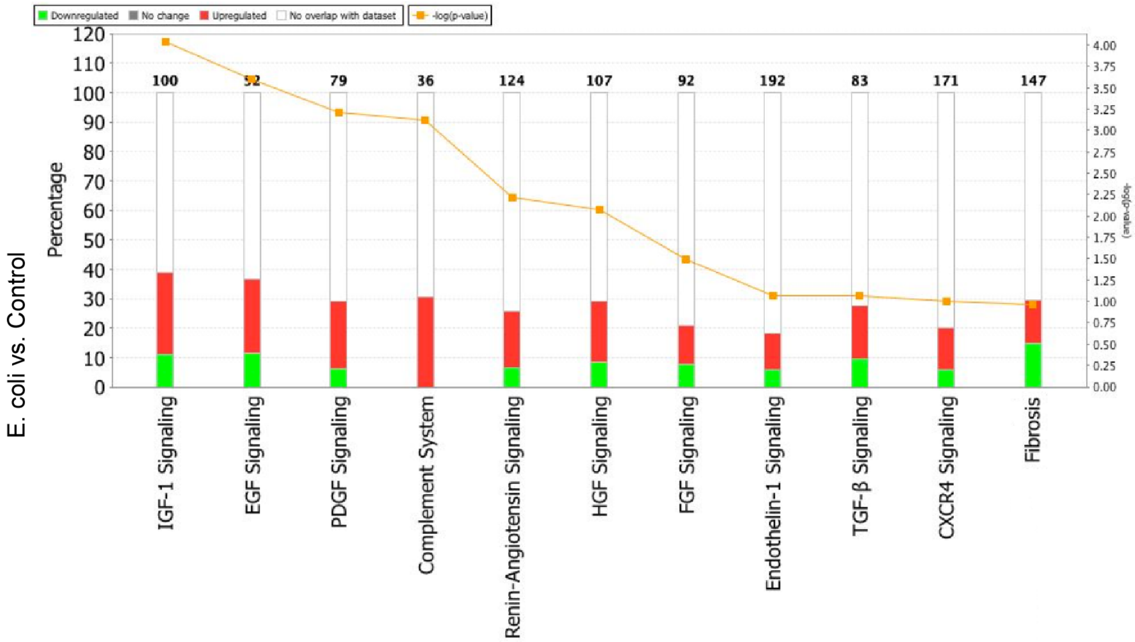

B

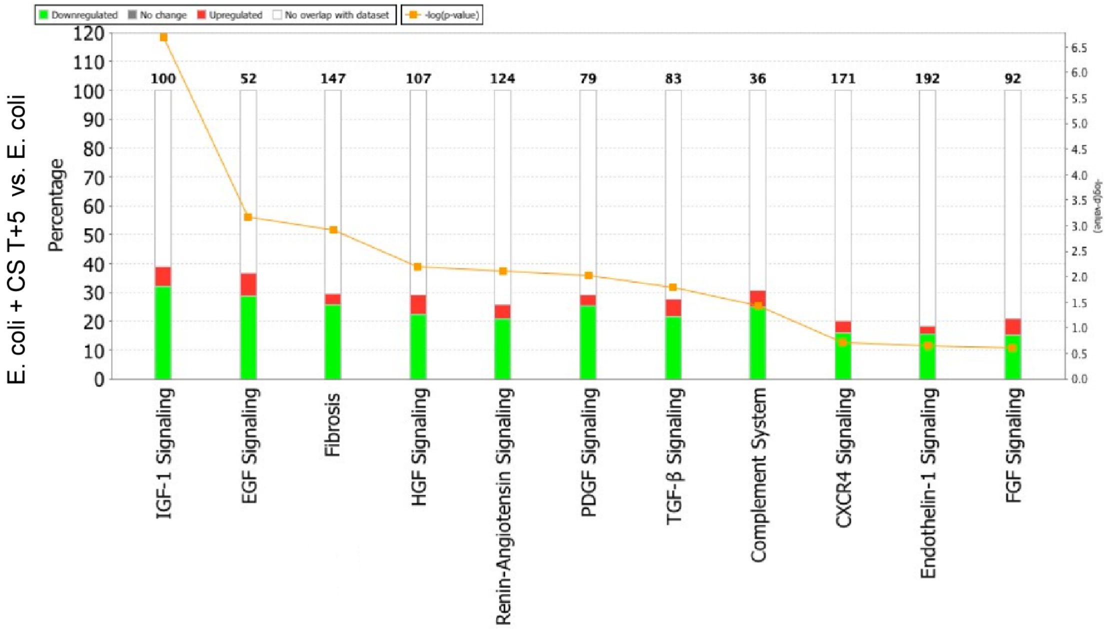

Figure E2: Canonical pathways up-regulated by sepsis (A) and down-regulated by compstatin treatment of septic baboons.

The stacked barchart displays the percentage of upregulated (red) and down-regulated (green) genes in each canonical pathway, out of the total molecules that make each pathway, shown above each column. Y axis shows the -log p-values, as calculated by Fisher's exact test right-tailed.

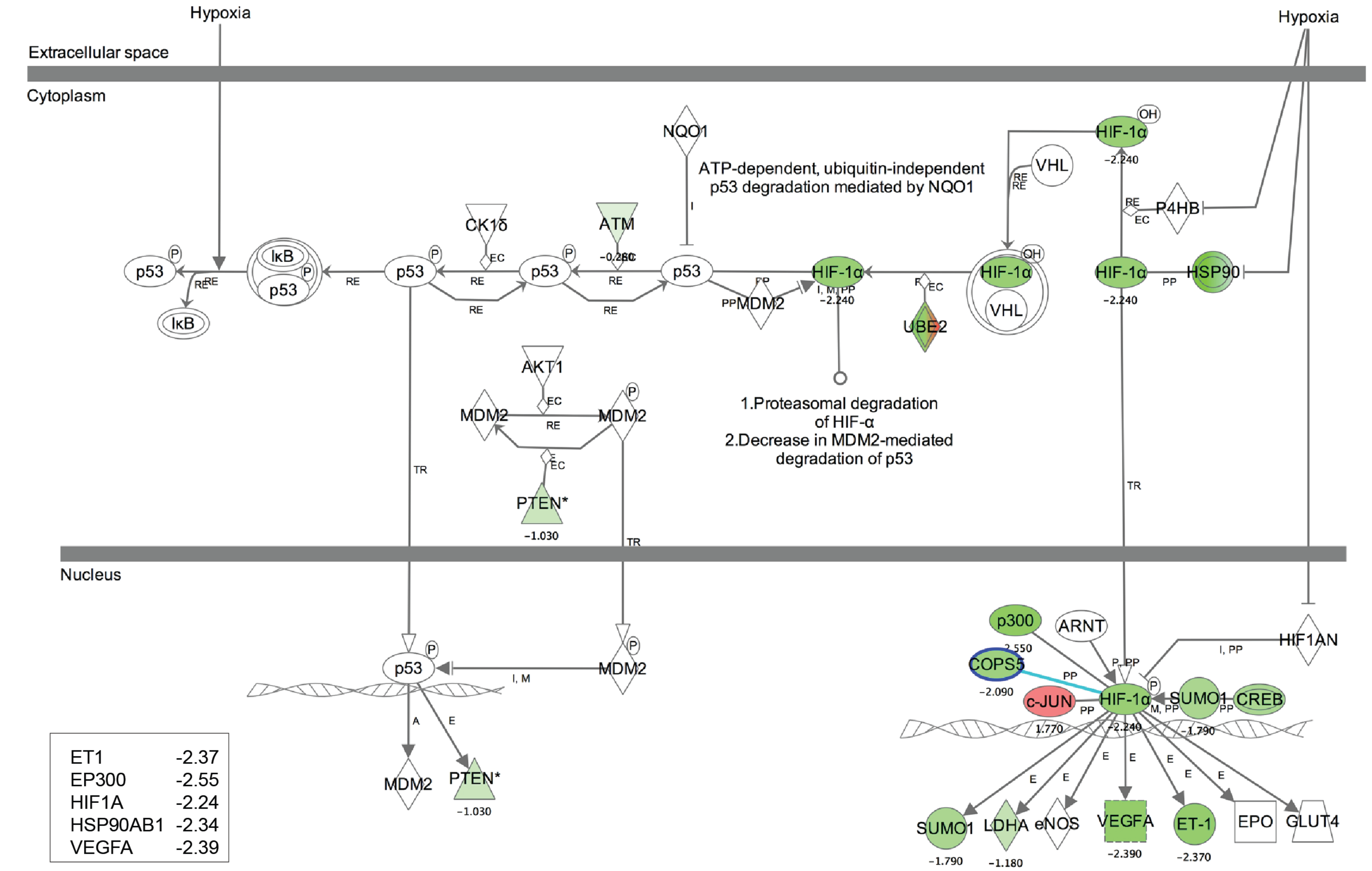

Figure E3: Compstatin treatment downregulates hypoxia inducible genes.
(HIF1a canonical pathway obtained by IPA shows that compstatin treatment during the second stage of E. coli sepsis (E coli +CS T + 5) leads to downregulation (green) of sepsis-induced hypoxia signaling genes, transcription factors and co-activators, including hypoxia controlled genes, like VEGFA and endothelin (ET-1).

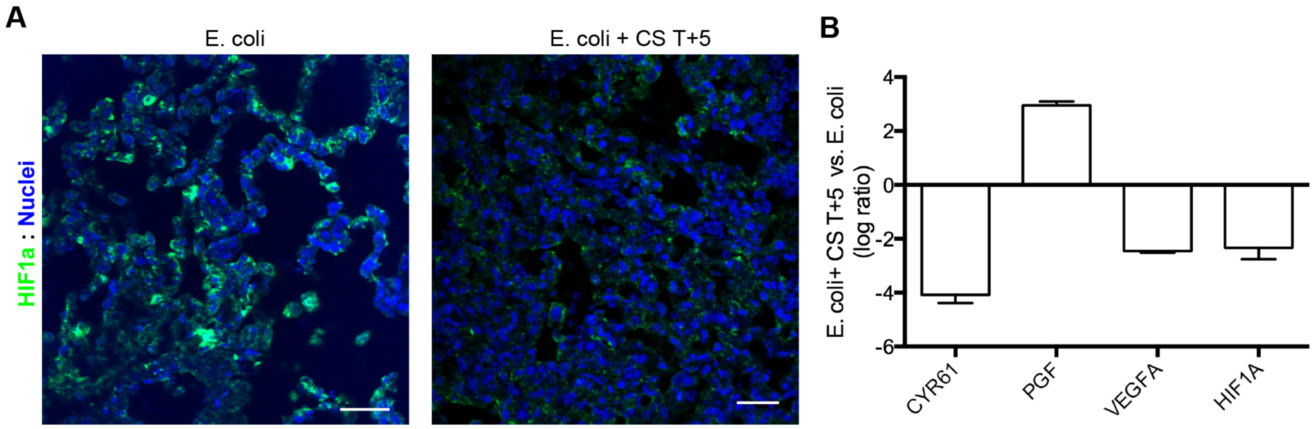

Figure E4: Compstatin treatment downregulates sepsis induced hypoxia.
(A): Immunofluorescence microscopy shows strong HIF1a staining in the lung of non-treated animals (E. coli) but not in the animals treated with compstatin during the second stage (E coli +CS T + 5). Magnification bar, 50µm. B: Log ratio representation of angiogenic proteins expression in compstatin treated group (E coli +CS T + 5) vs. non-treated septic animals (E. coli) shows that besides HIF1a, the angiogenic molecues VEGF and CYR61 are decreased while PGF expression is increased. Data are shown as log ratio mean ± SEM of 3 biological replicates.

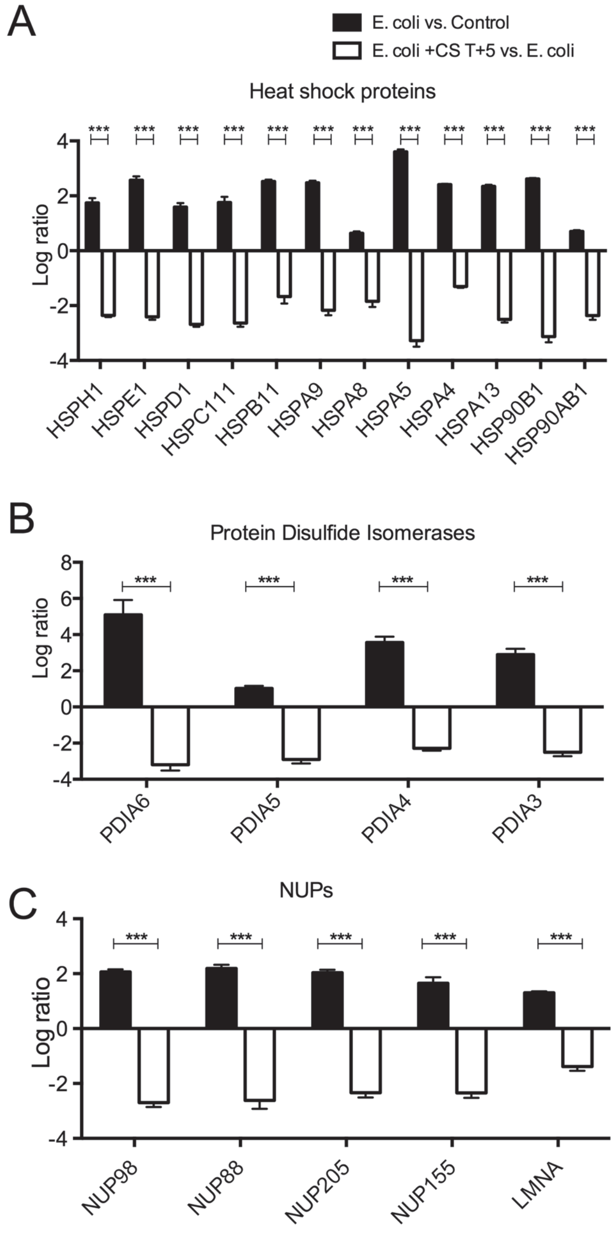

Figure E5: Expression of cellular stress response genes.
Log ratio representation of microarray data show that sepsis (E. coli vs.control) increased the expression of heat shock proteins (A), protein disulfide isomerases (B) and nuclear pore proteins (NUPs; C), while compstatin treatment consistently decreased these genes. Data are shown as log ratio mean ± SEM of 3 biological replicates. Multiple t test with Holm-Sidak multiple comparisons correction. ***P<0.001.

Cell proliferation

Comparison	Function / genes	P value	Number of genes
E. coli vs. Ctrl.	proliferation of cells: ↑ADAM10, ↑ADM, ↑ADORA2B, ↑AIF1, ↓ALMS1, ↑AMD1Δ, ↓ANGPT1, ↑ANXA1, ↑ATF3, ↑ATIC, ↓ATM, ↑B3GNT2, ↑BCAR3, ↑BCL2A1, ↑BCL6, ↑BCR, ↓BGN, ↓BMPR2, ↑BOP1, ↑C1QBP, ↑C3, ↓C5ORF13, ↓CADM1, ↑CALR, ↑CASP1, ↑CCDC92 (includes EG:80212), ↑CCL2, ↑CD14, ↑CD44, ↑CD86, ↑CDC27, ↓CDK19, ↓CDK6, ↑CDK7, ↑CFB, ↑CHST11, ↑CKS2, ↑COL4A1, ↓COL4A3, ↓COMT, ↑CSDA, ↑CSK, ↓CSNK1A1, ↑CSNK2A1, ↑CTSB, ↑CXCL10, ↑CXCL2, ↑CXCL9, ↑CYBB, ↑CYLD, ↑DAB2, ↓DACH1, ↓DDX11/DDX12, ↑DERL2, ↑DHCR24, ↑DIABLO, ↑DKC1, ↑DNAJA2, ↑EBNA1BP2, ↑EEF1E1, ↓EFNB2, ↑EIF4EBP1, ↑EMP1, ↓EPOR, ↑EPS8, ↑ETV6, ↑FASN, ↑FDFT1, ↓FGFR1, ↓FGFR3, ↑FKBP5, ↓FOLR1, ↑FOS, ↓FOXO1, ↑FPR2, ↓FUS, ↑FYB, ↓GAS6, ↓GATA1, ↓GATA2, ↑GBP1 (includes EG:2633), ↓GLP1R, ↓GNB1, ↑GNL3, ↓GPC3, ↑GPC4, ↑GRP, ↑HCK, ↑HCLS1, ↓HES1, ↓HEY1, ↑HIF1A, ↑HMGCR, ↑HMOX1, ↓HOXA5, ↑HPRT1, ↑HSP90B1, ↑HSPA5, ↑IDO1, ↑IFI30, ↑IFITM1, ↑IGF2, ↑IGFBP2, ↑IL15, ↑IL1RN, ↑IL4R, ↓IL6ST, ↓IL7R, ↑INSIG1, ↑IRF1, ↑ISG20, ↓ITCH, ↑ITGAM, ↑ITGB2, ↓JAG1, ↑JAK2, ↓JUN, ↑JUNB, ↑KCNA3, ↑KLF4, ↓KLF9, ↑LCN2, ↑LCP2, ↑LDHA, ↓LRP6, ↑LST1Δ, ↓LTBP4, ↑LYN, ↑MANF, ↓MAP2K7, ↓MAP3K3, ↑MAP4K1, ↑MAPK1, ↑MAPKAPK2, ↑MCFD2, ↑MCTS1, ↑MED7 (includes EG:9443), ↑MERTK, ↑MET, ↓MLL, ↑MMP9, ↑MORF4L1*, ↑MST4, ↑MUC1, ↑MVP, ↑MYC, ↓MYH10, ↑MYOF, ↑NBN, ↑NCF1, ↓NDE1, ↑NFE2, ↓NFIB, ↑NME1, ↑NOLC1, ↓NOX5, ↑NPM1 (includes EG:4869), ↑NR1H3, ↑NUP98Δ, ↑NUPR1, ↑PARK7, ↑PCNA, ↓PDPK1, ↑PDXKΔ, ↓PGF, ↑PGK1, ↑PIK3CB, ↑PIK3CG, ↓PIK3R1, ↑PIM1, ↑PITPNA, ↑PLA2G2A, ↑PLA2G4A, ↑PLAC8, ↑PLAT, ↑PLSCR1, ↑PNP, ↓PRDX2, ↑PRDX4, ↑PRKCB, ↑PRKDC, ↑PRMT5, ↑PSMC3, ↓PTGDS, ↑PTGER4, ↓PTPRC, ↑PTX3, ↑RAC2, ↑RARRES1, ↑RASGRF1, ↓RBM5, ↑RCAN1Δ, ↓RECK, ↑RELA, ↑RETN, ↑RHOG, ↑RIPK2, ↓RORA, ↑S100A8, ↑S100A9, ↑S100P, ↓SATB1, ↑SDC2, ↑SERPINE1, ↑SERPINE2, ↑SFN, ↑SH2B3, ↑SHMT2, ↑SIRPA, ↑SOCS3, ↑SOD2, ↑SOS1, ↑SOX17, ↓SOX4, ↓SPN, ↓SPTBN1, ↑SRGN, ↑SSR1, ↑STAM, ↑STAM2, ↓STARD13, ↑STAT1, ↑STC2, ↓TCF4, ↓TCF7, ↓TCF7L2, ↑TCIRG1, ↑TEAD4, ↓TERF1, ↑TFRC, ↑TGFBI, ↑TGM2, ↑THBS2, ↑THRA, ↓THY1, ↑TIAL1, ↓TIAM1, ↑TIMP1, ↑TLR2, ↑TNFRSF6B, ↓TPR, ↓TRA@, ↑TRIB1, ↑TRIM27, ↑TRRAP, ↑UBE2N, ↑UCHL1, ↑VCAM1, ↑VCAN, ↑VDAC1, ↓VIPR1, ↑WARS, ↑WIPF1, ↓WNK1Δ, ↑WTAP, ↓ZEB1, ↑ZNF259	2.67E-08	257
E. coli+CS T+5 vs. E. coli	proliferation of cells: ↓ABCB1, ↑ABCG1, ↓ABCG2, ↓ABI1, ↓ACVR1, ↑ACVRL1, ↓ADAM10, ↓ADAM17, ↓ADM, ↓ADORA2B, ↓ADRB2, ↓AGER, ↓AGPS, ↑AIF1, ↓AIM2, ↓AKAP13, ↓AKR1B1, ↓ALDH1A2, ↑ALMS1, ↓AMBRA1, ↓AMD1Δ, ↑ANGPT1, ↓ANP32A, ↓ANXA1, ↓ANXA7, ↓APOA1, ↓APOE, ↓APP, ↑ARHGEF1, ↓ASPH, ↓ATF3, ↓ATIC, ↓ATM, ↓ATP6AP1, ↓B3GNT2, ↓BAX, ↓BCAR3, ↓BCL2A1, ↓BCL2L1, ↓BCL3, ↓BCL6, ↓BCR, ↑BGN, ↑BID, ↓BLNK, ↑BMPR2, ↓BOP1/LOC727967, ↓BRD4, ↓BST2, ↓BTG3, ↓BUB3, ↓C1QBP, ↓C3, ↓C5, ↓C5orf13, ↑CADM1, ↓CALCRL, ↓CALR, ↓CAPRIN1, ↓CASP1, ↓CAT, ↓CAV1, ↓CCDC92 (includes EG:80212), ↓CCL2, ↓CCL21, ↓CCND1, ↓CCNG2, ↓CCT2, ↓CD14, ↓CD164, ↓CD36, ↓CD44, ↓CD47, ↑CD86, ↓CD9, ↓CDC16, ↓CDC25B, ↓CDC27, ↓CDC7, ↓CDK1, ↑CDK19, ↓CDK6, ↓CDK7, ↓CDKN1C, ↓CDKN2C, ↓CEBPB, ↓CEBPD, ↓CFB, ↓CFL1, ↑CFLAR, ↓CHST11, ↓CKS2, ↓CLU, ↓CNOT8, ↓COL18A1, ↓COL1A1, ↓COL4A1, ↓COL4A2, ↓COL4A3, ↑COMT, ↓COPS2, ↓COPS5, ↓CORO1A, ↓CREB1, ↓CREMΔ, ↓CRYAB, ↓CSDA, ↓CSE1L, ↓CSF1R, ↑CSF3R, ↓CSK, ↓CSNK1A1, ↓CSNK2A1, ↓CST6, ↓CTGF, ↓CTSB, ↓CUL2, ↑CUL7, ↓CXADR, ↓CXCL10, ↓CXCL12, ↓CXCL2, ↓CXCL9, ↓CXCR7, ↓CYBB, ↓CYLD, ↓CYR61, ↓DAB2, ↓DACH1, ↑DDX11/DDX12, ↓DDX17, ↓DERL2, ↓DHCR24, ↓DIABLO, ↑DIAPH1, ↓DKC1, ↑DLEC1, ↓DLG5, ↓DNAJA2, ↓DNAJB4, ↓DNMT1, ↓DPT, ↑DUSP1, ↑E2F4, ↓EBNA1BP2, ↓EDN1, ↓EDNRA, ↓EDNRB, ↓EED, ↓EEF1E1, ↑EFNB2, ↑EFS, ↓EGFR, ↓EGR1, ↓EIF4E, ↑EIF4EBP1, ↓ELAVL1, ↓ELF1, ↓ELF4, ↓ELN, ↓EMP1, ↓EMP2, ↓EMP3, ↓ENG, ↓ENPP2, ↓EP300, ↓EPCAM, ↑EPOR, ↓EPS15, ↓EPS8, ↓ETS2, ↓ETV6, ↑F2, ↓FABP4, ↓FABP5, ↓FASN, ↓FBLN5, ↓FCER1G, ↓FCGR2B, ↓FDFT1, ↑FGF1, ↑FGFR1, ↓FGFR2, ↑FGFR3, ↓FGL2, ↓FHL1, ↓FIGF, ↓FKBP1A, ↓FKBP5, ↓FLI1, ↓FLT1, ↓FN1, ↑FOLR1, ↓FOS, ↓FOSL2, ↑FOXF1, ↑FOXO1, ↓FOXO3, ↓FOXP1, ↑FPR2, ↑FURIN, ↑FUS, ↑FYB, ↓FYN, ↓FZD5, ↑GAS6, ↑GAS8, ↑GATA1, ↑GATA2, ↓GATA6, ↓GBP1, ↓GJA1, ↑GLI3, ↑GLP1R, ↓Gm5611, ↓GMNN, ↑GNB1, ↓GNL3, ↑GOLGA2, ↓GPC3, ↓GPC4, ↓GPI, ↓GPX1, ↓GRK5, ↓GRP, ↓GSK3B, ↑GSTM1, ↓GUCY1A3, ↓GUCY1B3, ↓HCK, ↓HCLS1, ↓HDAC4, ↓HES1, ↑HEY1, ↓HHEX, ↓HIF1A, ↓HIPK1, ↑HIPK2, ↓HK1, ↓HLA-DPB1, ↓HLA-DRB1, ↓HMGCR, ↓HMOX1, ↓HOXA5, ↓HPGD, ↓HPRT1, ↓HSP90B1, ↓HSPA5, ↓HSPD1, ↓ICAM1, ↓ID1, ↓ID3, ↓IDO1, ↓IFI30, ↓IFITM1, ↓IFITM3, ↓IGF1, ↓IGF1R, ↓IGF2, ↓IGF2R, ↓IGFBP2, ↓IGFBP4, ↓IGFBP5, ↓IGFBP6, ↓IGFBP7, ↓IGHM*, ↓IGKC*, ↑IKBKG, ↓IL13RA1, ↓IL15, ↓IL1R1, ↓IL1RN, ↓IL2RG, ↓IL4R, ↑IL6ST, ↑IL7R, ↓IMMT, ↓INHBA, ↑INO80B, ↓INSIG1, ↓IRF1, ↓IRF2, ↓ISG15, ↓ISG20, ↑ITCH, ↓ITGAM, ↓ITGB2, ↑ITGB4, ↑JAG1, ↓JAG2, ↓JAK2, ↑JUN, ↓JUNB, ↓JUP, ↓KCNA3, ↓KDR, ↓KIFAP3, ↓KIT, ↓KLF2, ↓KLF4, ↓KLF6, ↑KLF9, ↑KLRD1, ↓KRAS, ↓LAMA2, ↓LAMB1, ↓LASP1, ↓LCN2, ↓LCP2, ↓LDHA, ↓LGALS1, ↓LGALS3, ↓LIPA, ↓LOX, ↓LPIN1, ↓LRIG1, ↓LRP6, ↑LRRC32, ↑LST1Δ, ↑LTBP4, ↓LY86, ↓LYN, ↓MAGED1, ↓MANF, ↓MAP2K1, ↓MAP2K2, ↑MAP2K7, ↑MAP3K3, ↓MAP4K1, ↓MAPK1, ↓MAPK14, ↓MAPKAPK2, ↓MAPRE1, ↓MARCKSL1, ↓MAX, ↓MBD2, ↓MCFD2, ↓MCTS1, ↓MED1, ↓MED7 (includes EG:9443), ↓MERTK, ↓MET, ↓MFN2, ↓MGP, ↓MIF, ↓MLL, ↓MMP14, ↑MMP19, ↓MMP2, ↑MMP9, ↓MORF4L1*, ↓MST4, ↓MT2A, ↓MTA1, ↓MUC1, ↓MVP, ↓MYC, ↓MYH10, ↓MYOF, ↓NAMPT, ↓NBN, ↑NCF1, ↓NCK1, ↓NCOA1, ↑NDE1, ↓NDRG2, ↓NF1, ↑NFE2, ↓NFIB, ↓NME1, ↓NOC3L, ↓NOLC1, ↓NOTCH3, ↑NOX5, ↓NR1H2, ↓NR1H3, ↓NR4A3, ↓NRP1, ↓NUP98Δ, ↓NUPR1, ↓PARK7, ↓PAXIP1, ↓PBX1, ↓PCGF2, ↓PCNA, ↓PDCD10, ↑PDPK1, ↓PDS5B, ↓PDXKΔ, ↓PDZK1IP1, ↓PEA15, ↓PECAM1, ↓PELO, ↑PF4, ↓PFKFB3, ↑PGF, ↓PGK1, ↓PIK3CB, ↓PIK3CG, ↑PIK3R1, ↓PIM1, ↓PITPNA, ↓PLA2G2A, ↓PLA2G4A, ↑PLA2G6, ↓PLAC8, ↓PLAGL1, ↓PLAT, ↓PLSCR1, ↓PMP22, ↓PNP, ↓PPARG, ↓PPM1D, ↑PRDX2, ↓PRDX4, ↓PRKAR1A, ↓PRKCB, ↓PRKD1, ↓PRKDC, ↓PRMT5, ↓PRNP, ↓PSEN1, ↓PSMC3, ↑PTAFR, ↓PTCH1, ↓PTEN*, ↑PTGDS, ↑PTGER1, ↓PTGER4, ↓PTK2, ↓PTP4A1Δ, ↓PTPN2, ↑PTPRC, ↓PTPRE, ↓PTX3, ↓PURA, ↑PYY, ↓RAC2, ↓RALA, ↓RAP1B, ↓RAPGEF2, ↓RARA, ↓RARRES1, ↓RASGRF1, ↓RB1, ↓RBBP4, ↓RBM5, ↓RBM9, ↑RBP4, ↓RCAN1Δ, ↑RECK, ↓RELA, ↑RETN, ↓RGS2, ↓Rheb (rat), ↑RHOBTB2, ↓RHOG, ↓RIPK2, ↓RNF7, ↑RORA, ↓RPS6KA3, ↓RUNX1T1, ↓S100A11, ↑S100A8, ↑S100A9, ↑S100P, ↑S1PR1, ↓SAT1, ↑SATB1, ↓SDC2, ↑SERPINA1, ↓SERPINE1, ↓SERPINE2, ↑SET, ↓SFN, ↓SFTPD, ↑SH2B2, ↓SH2B3, ↑SH2D1A, ↓SHC1, ↓SHMT2, ↑SIGLEC7Δ, ↓SIRPA, ↓SKAP2, ↑SLAMF7, ↓SLC29A1, ↓SMAD2, ↓SMAD7, ↓SMARCA2, ↑SMO, ↓SNIP1, ↓SOCS2, ↓SOCS3, ↓SOD2, ↓SOS1, ↓SOX17, ↑SOX4, ↓SPARC, ↑SPN, ↓SPRY2, ↑SPTBN1, ↓SRGN, ↓SRSF2, ↓SSR1, ↓STAM, ↓STAM2, ↓STARD13, ↓STAT1, ↓STAT3, ↑STAT4, ↓STAT6, ↓STC2, ↑STK11, ↓SUMO1Δ, ↓SUMO3, ↓SUZ12*, ↓TACC2, ↓TACSTD2, ↓TAL1, ↓TAX1BP3, ↓TBC1D8, ↓TBX3, ↑TCF4, ↑TCF7, ↑TCF7L2, ↓TCIRG1, ↓TCP1, ↓TEAD4, ↑TERF1, ↑TES, ↓TFRC, ↓TGFB1I1, ↓TGFB2, ↓TGFBI, ↓TGIF1, ↓TGM2, ↑THBD, ↓THBS2, ↑THRA, ↑THY1, ↓TIAL1, ↓TIAM1, ↓TIMP1, ↓TIMP3, ↓TJP1, ↓TLR2, ↓TNFRSF21, ↓TNFRSF6B, ↓TNFSF13, ↑TPR, ↑TPSAB1, ↑TRA@, ↓TRIB1, ↓TRIM27, ↓TRIO, ↓TRRAP, ↓TSC22D3Δ, ↑TSPAN32, ↑TSPY1Δ, ↓TXNIP, ↓TXNRD1, ↓UBE2A, ↓UBE2N, ↓UBR5, ↓UCHL1, ↑UGCG, ↓VCAM1, ↓VCAN, ↓VDAC1, ↓VEGFA, ↓VIM, ↑VIPR1, ↓VWF, ↓WARS, ↓WFDC1, ↓WIPF1, ↑WNK1Δ, ↓WNT5A, ↓WTAP, ↓YBX1, ↑YY1, ↓ZBTB16, ↑ZEB1, ↓ZNF259	5.83E-13	584

Fibroblast proliferation

Comparison	Function / genes	P value	Number of genes
E. coli vs. Ctrl.	proliferation of fibroblast cell lines: ↑AMD1Δ, ↑ATIC, ↑BOP1, ↓C5ORF13, ↑CKS2, ↑CXCL10, ↓DACH1, ↑EBNA1BP2, ↑ETV6, ↓FOXO1, ↑GRP, ↑HMOX1, ↑IGF2, ↓JUN, ↑JUNB, ↑LDHA, ↓MAP2K7, ↑MAPK1, ↑MAPKAPK2, ↑MERTK, ↑MET, ↑MYC, ↑NPM1 (includes EG:4869), ↑PLAC8, ↑PLSCR1, ↑S100P, ↑SOS1, ↑TGM2, ↓TPR, ↓WNK1Δ	4.80E-03	30
E. coli+CS T+5 vs. E. coli	proliferation of fibroblast cell lines: ↓AGER, ↓AKR1B1, ↓AMD1Δ, ↓ATIC, ↓BAX, ↓BCL2L1, ↓BOP1/LOC727967, ↓BTG3, ↓C5orf13, ↓CAPRIN1, ↓CAT, ↓CAV1, ↓CCND1, ↓CD9, ↓CEBPB, ↓CKS2, ↓COL1A1, ↓COPS2, ↓COPS5, ↓CSF1R, ↑CUL7, ↓CXCL10, ↓CXCL12, ↓CXCR7, ↓DACH1, ↓EBNA1BP2, ↓ETV6, ↑FGF1, ↓FN1, ↑FOXO1, ↓Gm5611, ↓GRK5, ↓GRP, ↑HIPK2, ↓HMOX1, ↓IGF1, ↓IGF2, ↑JUN, ↓JUNB, ↓KLF2, ↓KLF6, ↓LDHA, ↓LOX, ↑MAP2K7, ↓MAPK1, ↓MAPK14, ↓MAPKAPK2, ↓MERTK, ↓MET, ↓MIF, ↓MYC, ↓NOC3L, ↓PBX1, ↓PLAC8, ↓PLSCR1, ↓PPARG, ↓PTEN*, ↓PTK2, ↓PTPN2, ↓RB1, ↓RPS6KA3, ↑S100P, ↑SH2B2, ↓SOS1, ↓STAT3, ↓SUZ12*, ↓TGIF1, ↓TGM2, ↑TPR, ↓TSC22D3Δ, ↓TXNIP, ↑WNK1Δ	5.56E-06	72

Figure E6-7: Lists of pathway-specific differential expressed genes involved in cell proliferation (E6), and migration/chemotaxis (E7). IPA functional analysis demonstrates that E. coli sepsis strongly increased the number of upregulated transcripts of genes involved in cell migration, cell spreading, fibroblast transformation and cell proliferation (E. coli vs. Controls; red arrows), while compstatin treatment down-regulated the genes involved in these functions (E. coli + CS T+5 vs. E. coli; green arrows). P-values and number of genes that are differen-ntially expressed in each dataset are shown. Right-tailed Fisher's exact test was used to calculate a p-value determining the probability that each biological function and/or disease assigned to that data set is due to chance alone.

A. Chemotaxis

Comparison	Category / genes	P value	Number of genes
E. coli vs. Ctrl.	chemotaxis: ↓ADRBK1, ↓ANGPT1, ↑ANXA1, ↑BCR, ↑C3, ↑CALR, ↑CCL2, ↑CD44, ↓COL4A3, ↑CSK, ↑CX3CL1, ↑CXCL10, ↑CXCL2, ↑CXCL9, ↑DARC, ↓FGFR1, ↑FGR, ↑FPR2, ↓GAS6, ↓GNB1, ↑HCK, ↑HP*, ↑IL15, ↑ITGAM, ↑ITGB2, ↓JUN, ↑LYN, ↑MAPK1, ↑MAPKAPK2, ↑MMP9, ↓PGF, ↑PIK3CB, ↑PIK3CG, ↑PPIB, ↑PRKCB, ↓PTPRC, ↑RAC2, ↓RALBP1, ↑S100A8, ↑S100A, ↑SERPINA3, ↑SIRPA, ↑SOCS3, ↓SPN, ↑THBS2, ↓TIAM1, ↑TLR2, ↑TNFRSF6B, ↑TRIB1, ↑TYMP, ↓VIPR1, ↑YARS	5.00E-03	52
E. coli+CS T+5 vs. E. coli	chemotaxis: ↑ADRBK1, ↓AGER, ↑ANGPT1, ↓ANXA1, ↓APP, ↓BCR, ↓C3, ↓C5, ↓CALR, ↓CAV1, ↓CCL2, ↓CCL21, ↓CCND1, ↓CD36, ↓CD44, ↓CD47, ↓COL4A3, ↓CORO1A, ↑CSF3R, ↓CSK, ↓CX3CL1, ↓CX3CR1, ↓CXCL10, ↓CXCL12, ↓CXCL14, ↓CXCL2, ↓CXCL9, ↓CXCR7, ↓CYR61, ↓DARC, ↑DIAPH1, ↓EDN1, ↓EDNRB, ↓EGFR, ↓ELN, ↓ENPP2, ↑F2, ↓FCER1G, ↓FCGR2A, ↑FGFR1, ↓FGR, ↓FIGF, ↓FLT1, ↓FN1, ↑FPR1, ↑FPR2, ↓FYN, ↑GAS6, ↓GNAI3, ↑GNAO1, ↑GNB1, ↓HCK, ↓HEBP1, ↑HP*, ↓HSPD1, ↓IGF1, ↓IGF2R, ↓IL15, ↓IL1R1, ↓INHBA, ↓ITGAM, ↓ITGB2, ↑ITGB4, ↓JAG2, ↑JUN, ↓KAL1, ↓KDR, ↓KIT, ↓LGALS1, ↓LGALS3, ↑LILRA6Δ, ↓LOX, ↓LYN, ↑LYST, ↓MAP2K1, ↓MAPK1, ↓MAPK14, ↓MAPKAPK2, ↓MMP14, ↓MMP2, ↑MMP9, ↓NCK1, ↓NRP1, ↓PDE4B, ↑PF4, ↑PGF, ↓PIK3CB, ↓PIK3CG, ↑PLA2G6, ↓PLEC, ↓PPARG, ↓PPIB, ↓PRKCB, ↓PRNP, ↑PTAFR, ↓PTEN*, ↓PTK2, ↑PTPRC, ↓RAC2, ↓RALA, ↑RALBP1, ↑S100A4, ↑S100A8, ↑S100A9, ↑S1PR1, ↑SEMA3B, ↑SERPINA1, ↓SERPINA3, ↓SFTPD, ↓SIRPA, ↓SLIT2, ↓SOCS3, ↑SPN, ↓TGFB2, ↓THBS2, ↓TIAM1, ↓TLR2, ↓TNFRSF6B, ↓TRIB1, ↑TYMP, ↓VEGFA, ↑VIPR1, ↓YARS	1.98E-05	123

B. Cell migration

Comparison	Category / genes	P value	Number of genes
E. coli vs. Ctrl.	migration of cells: ↑ADAM10, ↑ADM, ↑ALOX5, ↓ANGPT1, ↑ANXA1, ↑BCAR3, ↑BCR, ↓BMPR2, ↑C3, ↓C5ORF13, ↑CALR, ↑CCL2, ↑CD44, ↑CD86, ↑CDK7, ↑COL4A1, ↓COL4A3, ↑CSK, ↑CTSB, ↑CX3CL1, ↑CXCL10, ↑CXCL2, ↑CXCL9, ↑DAB2, ↑DARC, ↓DCBLD2, ↑DDX3X, ↓EFNB2, ↓EPOR, ↑ETV6, ↓FGFR1, ↑FGR, ↑FHL2, ↓FOXO1, ↑FPR2, ↑FYB, ↓GAS6, ↓GATA1, ↑GRP, ↑HCK, ↓HEY1, ↑HIF1A, ↑HMGCR, ↑HMOX1, ↑HP*, ↑IGF2, ↑IGFBP2, ↑IL15, ↓IL6ST, ↑ITGAM, ↑ITGB2, ↓JAG1, ↑JAK2, ↓JUN, ↑KLF4, ↓LAMC2, ↑LCN2, ↓LRP6, ↓LTBP2, ↑LYN, ↑MAFB, ↑MAPK1, ↑MAPKAPK2, ↑MET, ↓MLL, ↑MMP9, ↓MRC2, ↑MUC1, ↑MYC, ↓MYH10, ↑MYO1F, ↑NCF1, ↓NDE1, ↑NME1, ↓OPHN1, ↓PALLD, ↓PDPK1, ↓PGF, ↑PIK3CB, ↑PIK3CG, ↓PIK3R1, ↑PIM1, ↑PLAT, ↑PPIB, ↑PRKCB, ↓PRKCI, ↑PTGER4, ↓PTPRC, ↑RAC2, ↓RALBP1, ↑RARRES1, ↑RASGRF1, ↑RASGRP2, ↓RECK, ↑RELA, ↓REST, ↑RETN, ↑RHOG, ↓ROCK2, ↑RRAS2, ↑S100A8, ↑S100A9, ↑S100P, ↑SDC2, ↑SEPT4Δ, ↑SERP1, ↑SERPINA3, ↑SERPINE1, ↑SERPINE2, ↑SIRPA, ↑SLC3A2, ↓SLIT3, ↑SOCS3, ↑SOD2, ↑SORT1, ↑SP100Δ, ↓SPN, ↑STAT1, ↓TCF7L2, ↑TGFBI, ↑TGM2, ↑THBS2, ↓THY1, ↓TIAM1, ↑TIMP1, ↑TLR2, ↑TNFRSF6B, ↑VCAM1, ↑VCAN, ↓VIPR1, ↑WARS, ↓WASL (includes EG:8976), ↑WDR44, ↑YARS, ↑YWHAE	1.80E-03	135
E. coli+CS T+5 vs. E. coli	migration of cells: ↓ACVR1, ↑ACVRL1, ↓ADAM10, ↓ADAM17, ↓ADAM19, ↓ADM, ↓ADRB2, ↓AGER, ↓ALDOA, ↓ALOX5, ↑ANGPT1, ↓ANKS1A, ↓ANXA1, ↓APOA1, ↓APOE, ↓APP, ↓ARF6, ↑ARHGEF1, ↓ARRB1, ↓BAX, ↓BCAR3, ↓BCR, ↑BMPR2, ↓C3, ↓C5, ↓C5orf13, ↓CALCRL, ↓CALR, ↓CAP1, ↓CAT, ↓CAV1, ↓CCL2, ↓CCL21, ↓CCND1, ↓CD36, ↓CD44, ↓CD47, ↑CD82, ↑CD86, ↓CD9, ↓CDH11, ↓CDK1, ↓CDK7, ↓CFL1, ↓CLCN3, ↓CLIC4, ↓CLU, ↓COL18A1, ↓COL1A1, ↓COL4A1, ↓COL4A2, ↓COL4A3, ↓CORO1A, ↓CSE1L, ↓CSF1R, ↑CSF3R, ↓CSK, ↓CST6, ↓CTGF, ↓CTSB, ↓CX3CL1, ↓CX3CR1, ↓CXCL10, ↓CXCL12, ↓CXCL14, ↓CXCL2, ↓CXCL9, ↓CXCR7, ↓CYR61, ↓CYTIP, ↓DAB2, ↓DARC, ↓DCBLD2, ↓DDX3X, ↑DIAPH1, ↓DOCK4, ↑DUSP1, ↓EDN1, ↓EDNRB, ↑EFNB2, ↑EFS, ↓EGFR, ↓EGR1, ↓ELN, ↓ENG, ↓ENPP2, ↓EPAS1, ↑EPOR, ↓ETV6, ↓EZR, ↑F2, ↓FBLN5, ↓FCER1G, ↓FCGR2A, ↑FGF1, ↑FGFR1, ↓FGR, ↓FHL1, ↓FHL2, ↓FIGF, ↓FLT1, ↓FN1, ↑FOXF1, ↑FOXO1, ↓FOXO3, ↑FPR1, ↑FPR2, ↑FURIN, ↑FYB, ↓FYN, ↑GAS6, ↑GATA1, ↓GJA1, ↑GLI3, ↓GNAI3, ↑GNAO1, ↑GOLGA2, ↓GRP, ↓GSK3B, ↓GUCY1A3, ↓GUCY1B3, ↓HCK, ↓HEBP1, ↑HEY1, ↓HHEX, ↓HIF1A, ↓HMGCR, ↓HMOX1, ↑HP*, ↓HSP90AB1, ↓HSPD1, ↓ICAM1, ↓ID1, ↓ID3, ↓IGF1, ↓IGF1R, ↓IGF2, ↓IGF2R, ↓IGFBP2, ↓IGFBP4, ↓IGFBP5, ↓IGFBP6, ↑IKBKG, ↓IL15, ↑IL6ST, ↓INHBA, ↓IQGAP1, ↓ITGAM, ↓ITGB2, ↑ITGB4, ↑JAG1, ↓JAK2, ↑JUN, ↓JUP, ↓KATNA1, ↓KDR, ↓KIT, ↓KLF2, ↓KLF4, ↓LAMA2, ↓LAMB1, ↓LAMC2, ↑LASP1, ↓LCN2, ↓LDLR, ↓LGALS1, ↓LGALS3, ↓LHX6, ↑LILRA6Δ, ↓LOX, ↓LRP6, ↑LTBP2, ↓LYN, ↑LYST, ↓MAFB, ↓MAP2K1, ↓MAP2K2, ↓MAPK1, ↓MAPK14, ↓MAPKAPK2, ↑MARCKS, ↓MAX, ↓MCAM, ↓MET, ↓MGP, ↓MIF, ↓MLL, ↓MMP14, ↑MMP19, ↓MMP2, ↑MMP9, ↑MRC2, ↓MTA1, ↓MUC1, ↓MYC, ↓MYH10, ↓MYH9, ↓MYO1F, ↑NBL1, ↑NCF1, ↓NCK1, ↑NDE1, ↓NF1, ↓NME1, ↓NR2F2, ↓NR4A2, ↓NRP1, ↑OPHN1, ↓PAK2, ↑PAK4, ↑PALLD, ↓PDE4B, ↑PDPK1, ↓PECAM1, ↑PF4, ↑PGF, ↓PHB (includes EG:5245), ↓PIK3C2A, ↓PIK3CB, ↓PIK3CG, ↑PIK3R1, ↓PIM1, ↑PLA2G6, ↓PLAT, ↓PLEC, ↓PLXNA1, ↓PMP22, ↓PPAP2B, ↓PPARG, ↓PPFIA1, ↓PPIB, ↓PRKAR1A, ↓PRKCB, ↑PRKCI, ↓PRKD1, ↓PRKD2, ↑PRNP, ↓PSEN1, ↓PTEN*, ↓PTGER4, ↓PTK2, ↓PTP4A1Δ, ↓PTPN12, ↓PTPN2, ↑PTPRC, ↓RAC2, ↑RALBP1, ↑RAMP2, ↓RANBP9, ↓RAP1B, ↓RARRES1, ↓RASGRF1, ↓RASGRP2, ↑RECK, ↓RELA, ↑REST, ↑RETN, ↓RHOG, ↑ROCK2, ↓RRAS2, ↓RTN4, ↑S100A4, ↑S100A8, ↑S100A9, ↑S100P, ↑S1PR1, ↓SARS, ↓SDC1, ↓SDC2, ↓SDCBP, ↓SELP, ↓SEMA5A, ↓SEPT4Δ, ↓SERP1, ↑SERPINA1, ↓SERPINA3, ↑SERPINB3, ↓SERPINE1, ↓SERPINE2, ↓SFTPD, ↓SHC1, ↓SIRPA, ↑SLC3A2, ↓SLIT2, ↑SLIT3, ↓SMAD7, ↑SMO, ↓SOCS3, ↓SOD2, ↓SORT1, ↓SP100Δ, ↓SPARC, ↑SPDEF, ↑SPN, ↓SPRY2, ↓STAT1, ↓STAT3, ↑STK11, ↑TCF7L2, ↓TDP2, ↓TGFB2, ↓TGFBI, ↓TGM2, ↓THBS2, ↑THY1, ↓TIAM1, ↓TIE1, ↓TIMP1, ↓TIMP3, ↓TLR2, ↓TNFRSF21, ↓TNFRSF6B, ↓TNFSF13, ↓TNS1, ↓TPM1, ↓TRIO, ↓VCAM1, ↓VCAN, ↓VCL, ↓VEGFA, ↑VIPR1, ↑VSNL1, ↓VWF, ↓WARS, ↑WASL (includes EG:8976), ↓WDR44, ↓WNT5A, ↓YARS, ↓YWHAE, ↓ZBTB16	9.36E-08	330

Figure E7

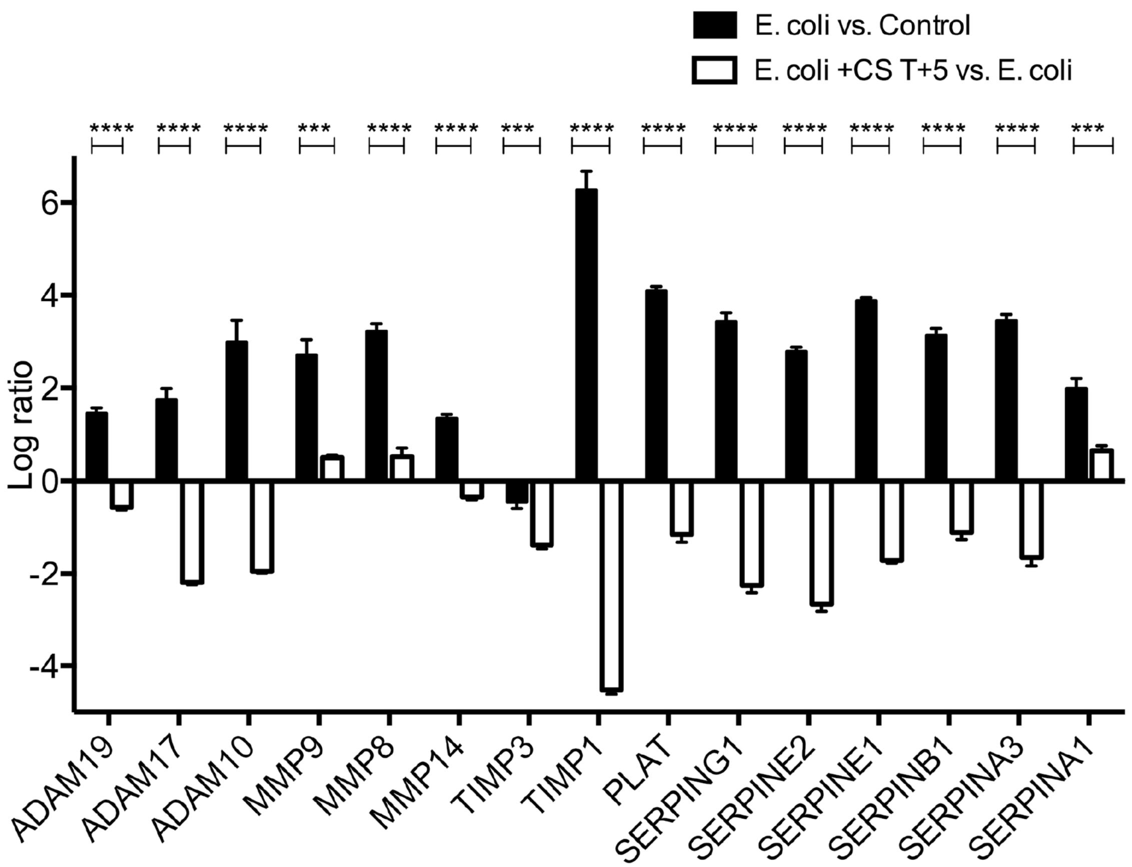

Figure E8: Expression of genes involved in extracellular matrix remodeling.
Log ratio of microarray data showing that compstatin treatment downregulated the expression of extracellular proteases and serine protease inhibitors induced by E. coli sepsis. Data are shown as log ratio mean±SEM of 3 biological replicates. Multiple t test with Holm-Sidak multiple comparisons corection. ***P<0.001; ****P<0.001.

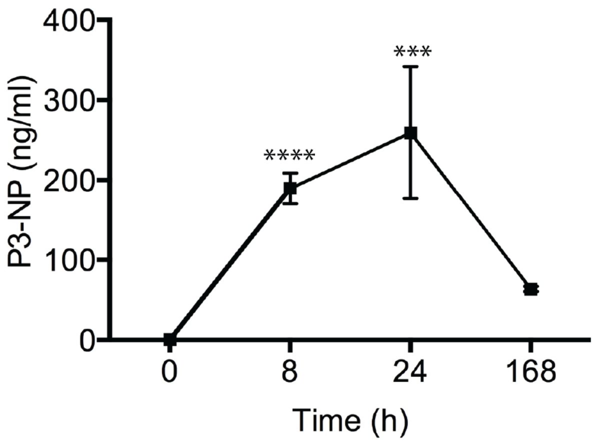

Figure E9: Time course of procollagen 3 N-terminal peptide levels in plasma of baboons challenged with LD50 *E. coli*.
Data are shown as mean±SEM; n=4 biological replicates per time-point; One-way ANOVA with Dunnett multicomparison test; ****P <0.0001; ***P <0.001 as compared to T0 group.
